# Mapping oto-pharyngeal development in a human inner ear organoid model

**DOI:** 10.1242/dev.201871

**Published:** 2023-10-05

**Authors:** Matthew R. Steinhart, Wouter H. van der Valk, Daniel Osorio, Sara A. Serdy, Jingyuan Zhang, Carl Nist-Lund, Jin Kim, Cynthia Moncada-Reid, Liang Sun, Jiyoon Lee, Karl R. Koehler

**Affiliations:** ^1^ Department of Otolaryngology, Boston Children's Hospital, Boston, MA 02115, USA; ^2^ F. M. Kirby Neurobiology Center, Boston Children's Hospital, Boston, MA 02115, USA; ^3^ Department of Otolaryngology-Head and Neck Surgery, Indiana University School of Medicine, Indianapolis, IN 46202, USA; ^4^ Medical Neuroscience Graduate Program, Indiana University School of Medicine, Indianapolis, IN 46202, USA; ^5^ Department of Otolaryngology-Head and Neck Surgery, Harvard Medical School, Boston, MA 02115, USA; ^6^ OtoBiology Leiden, Department of Otorhinolaryngology and Head & Neck Surgery; Leiden University Medical Center, Leiden 2333 ZA, the Netherlands; ^7^ The Novo Nordisk Foundation Center for Stem Cell Medicine (reNEW); Leiden University Medical Center, Leiden, 2333 ZA, the Netherlands; ^8^ Research Computing, Department of Information Technology; Boston Children's Hospital, Boston, MA 02115, USA; ^9^ Program in Neuroscience, Harvard Medical School, Boston, MA 02115, USA; ^10^ Department of Plastic and Oral Surgery, Boston Children's Hospital, Boston, MA 02115, USA; ^11^ Speech and Hearing Bioscience and Technology (SHBT) Graduate Program, Harvard Medical School, Boston, MA 02115, USA

**Keywords:** Inner ear, Single-cell genomics, Pluripotent stem cells, Organoids, Cell differentiation

## Abstract

Inner ear development requires the coordination of cell types from distinct epithelial, mesenchymal and neuronal lineages. Although we have learned much from animal models, many details about human inner ear development remain elusive. We recently developed an *in vitro* model of human inner ear organogenesis using pluripotent stem cells in a 3D culture, fostering the growth of a sensorineural circuit, including hair cells and neurons. Despite previously characterizing some cell types, many remain undefined. This study aimed to chart the *in vitro* development timeline of the inner ear organoid to understand the mechanisms at play. Using single-cell RNA sequencing at ten stages during the first 36 days of differentiation, we tracked the evolution from pluripotency to various ear cell types after exposure to specific signaling modulators. Our findings showcase gene expression that influences differentiation, identifying a plethora of ectodermal and mesenchymal cell types. We also discern aspects of the organoid model consistent with *in vivo* development, while highlighting potential discrepancies. Our study establishes the Inner Ear Organoid Developmental Atlas (IODA), offering deeper insights into human biology and improving inner ear tissue differentiation.

## Teaser

**Highlighted Article:** An *in vitro* model of human inner ear organogenesis created using pluripotent stem cells and analyzed using single-cell RNA sequencing to generate the Inner ear Organoid Developmental Atlas (IODA).

## INTRODUCTION

Inner ear dysfunction leading to deafness and balance disorders is a significant global health burden (Chadha et al., 2021). However, routine use of patient-derived inner ear explants for research is challenging due to the invasive and destructive nature of inner ear biopsy (Taylor et al., 2015). An *in vitro* culture system whereby researchers can derive inner ear tissue from human pluripotent stem cells would provide a platform to model human auditory and vestibular pathologies, to investigate developmental questions concerning inner ear organogenesis, and to evaluate promising therapeutics for human applications (Bermingham-McDonogh et al., 2012). An important goal has been to better define the chemical and physical signals required to generate *in vivo*-like functional inner ear tissue in an *in vitro* culture system to supplement the current animal models of the zebrafish, chick and mouse.

Past studies with animal models have developed a solid framework for understanding the development of the inner ear and its associated tissues. Inner ear development begins with the otic placode: a thickening of surface ectoderm adjacent to the hindbrain. The otic placode gives rise to the otic vesicle and, in turn, the tissues of the inner ear, including otic epithelium, hair cells and neurons. Along with the inner ear, other sensory structures of the head arise from the surface ectoderm bordering the neuroectoderm, collectively known as the cranial placodes (Baker and Bronner-Fraser, 2001; Schlosser, 2006). The cranial placodes comprise two sets of structures: those derived from posterior pre-placodal ectoderm, such as the otic and epibranchial placodes; and those derived from anterior pre-placodal ectoderm, such as the olfactory, lens and trigeminal placodes. As recently reviewed, these structures develop in close proximity and interact with each other (Koontz et al., 2023). These interactions are essential for advancing placode and sensory development; examples for the inner ear are the significant morphogenic contribution of neuroectoderm in specifying the otic placode (Ohyama et al., 2006), as well as the formation and fusion of neural crest cells forming glial cells of the cochleovestibular ganglion and melanocyte-like intermediate cells of the cochlear stria vascularis (Ritter and Martin, 2018). Therefore, cranial sensory development is a complex and multifaceted process, with the formation and interaction of multiple cell types and signaling pathways. Understanding the intricacies of this process is important for developing potential treatments for a range of developmental disorders affecting the inner ear and other cranial sensory structures.

Our group has recently developed a multi-stage 3D culture system that captures essential elements of inner ear development, including induction of surface ectoderm and cranial placodes (Koehler et al., 2017). Although there are other *in vitro* systems of inner ear tissue culture, we chose to employ our inner ear organoid system in this study because it captures key morphological changes observed during mouse and human development (Groves and Fekete, 2012; van der Valk et al., 2021). Organoid differentiation begins with the modulation of transforming growth factor (TGF), bone morphogenetic protein (BMP) and fibroblast growth factor (FGF) signaling pathways to induce the necessary progenitor cell types. Next, activation of the WNT signaling pathway induces otic placode tissue and, in turn, otic vesicles. The otic vesicles then self-organize and mature into elaborate multi-chambered cysts containing sensory epithelium with hair cells. Simultaneously, neurons establish a sensorineural network containing synaptic connections with organoid hair cells. Together with the otic tissue, hair-bearing skin and cartilage are produced (Koehler et al., 2017; Lee et al., 2020), suggesting that a collection of tissues reflecting a broader cranial region emerge in these cultures.

Inner ear organoids are a promising *in vitro* model for studying human auditory and vestibular pathologies. Yet it is crucial to continually optimize the approach to ensure its accurate representation of the native tissue (Nist-Lund et al., 2022). Despite significant progress, questions still need to be answered regarding the functionality and completeness of the organoids, underscoring the need to refine our techniques and tools to better understand their molecular and cellular properties. To this end, we employed single-cell transcriptomics, immunohistochemistry and live-cell imaging to meticulously capture a high-resolution time course of developing inner ear organoids, including associated mesenchymal and neural-glial cell types, which have been poorly characterized to date. Our findings reveal previously unrecognized cell lineages and provide insights into the tissue response to exogenous and endogenous signaling.

## RESULTS

### scRNA-seq data overview: diverse ectodermal subtypes emerge in inner ear organoid culture

**Fig. 1.**
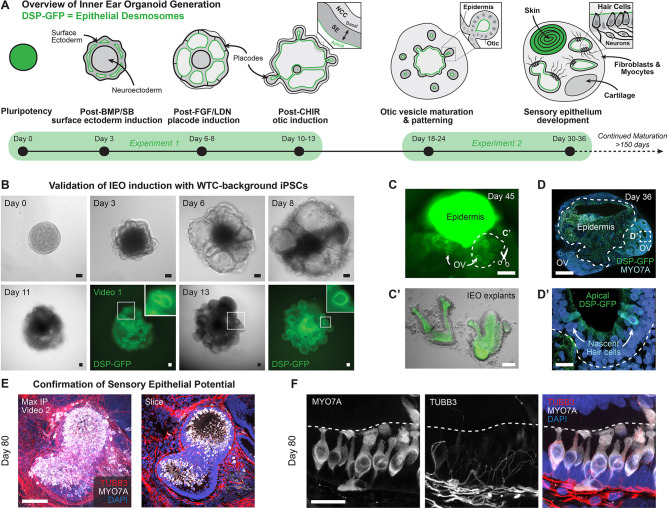
**Generating DSP-GFP inner ear organoids for single-cell sequencing.** (A) Overview of the process of guided differentiation of inner ear organoids, including time points used for experimental analysis. (B) Representative bright-field and fluorescent images of DSP-GFP hiPSC cell line differentiation. GFP fluorescence at day 11 and day 13 indicates expression of desmoplakin (DSP) in the epithelium contained in a developing inner ear organoid. Scale bars: 100 μm. See also Movie 1. (C,C′) DSP-GFP expression throughout a day 45 organoid with intense DSP-GFP signal in the epidermis (C) and retained DSP-GFP expression in developing otic vesicles and explanted otic vesicles (C′). Scale bars: 200 μm. (D) Overview of a day 36 inner ear organoid showing DSP-GFP signal and presence of MYO7A^+^ hair cells in otic vesicles. Scale bars: 200 μm. (D′) Higher magnification of the area outlined in D. Scale bars: 25 μm. (E) More mature, day 80, inner ear vesicles within an inner ear organoid containing MYO7A^+^ hair cells and surrounded by TUBB3^+^ neurons. Scale bar: 100 μm. See also Movie 2. (F) Detailed view of an inner ear vesicle in a late-stage inner ear organoid, day 80, containing MYO7A^+^ hair cells and TUBB3^+^ neurons that protrude towards the hair cells. Scale bar: 25 μm.

Since we first reported our human inner ear organoid induction system (Koehler et al., 2017), we have made several technical improvements to enhance the consistency and usability of the method across cell lines (see the Technical Notes section of the supplementary Materials and Methods) (Doda et al., 2023; Lee et al., 2022; van der Valk et al., 2023; Zhang et al., 2021). To better define the cell populations that arise during inner ear organoid differentiation, we performed single-cell RNA-sequencing (scRNA-seq) at multiple time points during development. Starting with aggregated pluripotent cells (day 0), we captured transcripts from differentiating cells of aggregates every 2-6 days until shortly after the emergence of hair cells (day 36) (Fig. 1A). Owing to the extensive coverage of numerous time points, we focused our scRNA-seq analysis on a single representative cell line that is commonly used in our lab: a gene-edited variant of the wild-type C (WTC; WTC-11) human induced pluripotent stem cell (hiPSC) line containing a GFP-tagged Desmoplakin (*DSP*) gene (Miyaoka et al., 2014; Roberts et al., 2017). DSP is involved in cell-cell adhesion by formation of desmosomes, and we could confirm DSP-GFP expression, which emerged concurrently with the differentiation of surface ectoderm and continued through cranial placode specification (Fig. 1B). DSP-GFP expression localized to the apical surface of epithelial cells and was maintained during otic placode and vesicle formation (Movie 1). This epithelial expression pattern of DSP-GFP remained during organoid development and maturation (Fig. 1C). Thus, the GFP reporter in this cell line was excellent for tracking organoid development by live-cell imaging to assess batch quality.

**Fig. 2.**
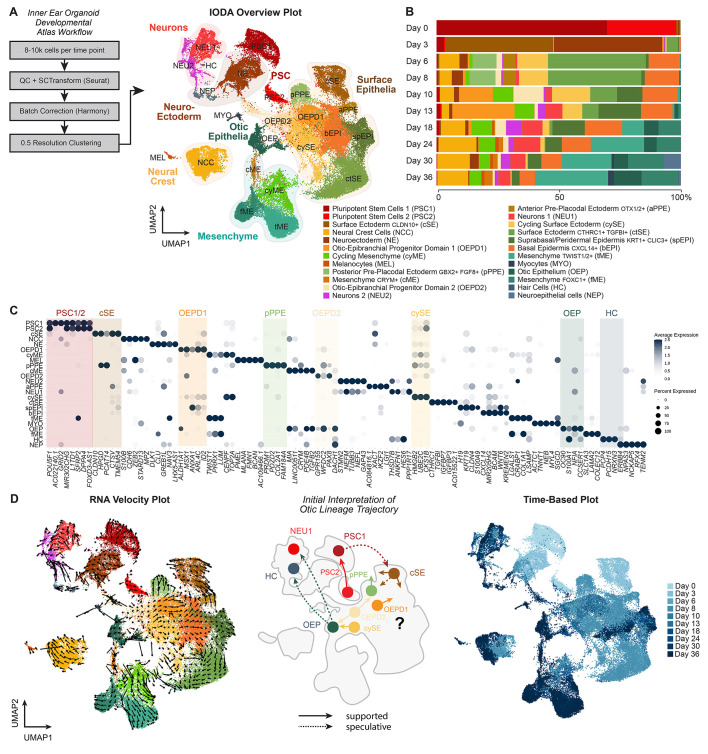
**The Inner Ear Organoid Developmental Atlas.** (A) Experimental design for generating the Inner Ear Organoid Developmental Atlas (IODA) showing the UMAP plotting the day 0 to day 36 integrated dataset with cell type annotations. (B) The relative contribution per cell type per included time point. (C) Dot plot of highly differentially expressed genes between clusters. (D) RNA velocity and time-based analysis on the day 0 to day 36 integrated IODA dataset. For the inferred otic development trajectory, see also Figs S1 and S2.

Using immunostaining, we identified MYO7A^+^ hair cells in organoids as early as day 36 of differentiation (Fig. 1D), with more pronounced sensory epithelial development by day 80 of differentiation and beyond (Fig. 1E-F, Movie 2). We sampled ten time points from day 0 (pluripotency) to day 36 (just after hair cell induction) with the intent of capturing the otic lineage specification process that eventually leads to a more mature phenotype as described previously (Doda et al., 2023; Koehler et al., 2017; van der Valk et al., 2023). We intended to obtain sufficient coverage to elucidate most cell lineage specification events. Therefore, we conducted two separate experiments to collect the samples (experiment 1 for days 0, 3, 6, 8, 10, 13; experiment 2 for days 18, 24, 30, 36) (Fig. 1A). We designated a shorter interval between each collection for the earlier time points since rapid changes in gene expression occur during the early stages of differentiation. Gene expression matrices were preprocessed, batch-corrected, and integrated using Seurat and Harmony software packages (Fig. 2A; see Materials and Methods section for detailed procedure) (Butler et al., 2018; Korsunsky et al., 2019). Principal component analysis (PCA) and uniform manifold approximation and projection (UMAP) were used to visualize cell clusters. To arrive at a ground truth UMAP plot, we varied the PC number (10, 30, 50 and 100) and then evaluated a test set of genes known to be expressed in the organoid model (see Quality Control section of the supplementary Materials and Methods). The 100 PC plot was selected based on clear clustering of otic lineage cells and hair cells. In addition, we evaluated *MKI67* expression, among other cell cycle-associated genes, to show that most clusters were not defined solely by cycling identity (Fig. S1A). The integrated data are available for exploration on the gene Expression Analysis Resource (gEAR) portal at https://umgear.org/p?l=InnerEarOrganoidAtlas.

We initially focused on defining the significant cell populations and their trajectories. Exploring the integrated 2D and 3D UMAP plots, we identified hiPSCs (PSC1 and PSC2: *POU5F1*, *NANOG*, *FOXH1*, *L1TD1*, *DPPA4* and *TERF1*), surface ectoderm cells (ctSE, cSE and cySE: *TFAP2C*, *EPCAM*, *CDH1*, *WNT6*, *KRT8*, *KRT18*, *KRT19*, *MSX1*, *MSX2* and *DLX5*), neuroectoderm cells (NE: *PAX6*, *ZIC2*, *ZIC5*, *OTX1*, *OTX2*, *TPBG* and *LHX5*), mesenchymal cells (cyME, tME, fME and cME: *TWIST1*, *VIM*, *PRRX1*, *LUM*, *COL1A1*, *COL3A1*, *COL5A1* and *EMP3*), neurons (NEU1, NEU2 and NEP: *STMN2*, *NEFM*, *NEFL*, *SYP*, *ELAVL2*, *ELAVL4*, *INSM1*, *NHLH1* and *DCX*), neural crest cells (NCC: *MPZ*, *S100B*, *SOX10*, *FOXD3*, *CDH19*, *ERBB3*, *PLP1* and *CDH6*), (pre)placodal cells (aPPE, pPPE and otic-epibranchial progenitor domain cells (OEPD1 and OEPD2): *OTX1*, *OTX2*, *GBX2* and *PAX8*), otic cells (OEP: *OC90*, *TBX2*, *OTOL1*, *USH1C*, *EMX2*, *PAX2*, *FGF9*, *SCX* and *OTOGL*), hair cells (HC: *PCP4*, *OTOF*, *USH2A*, *NRXN3*, *HES6* and *PCDH15*), epidermal cells (bEPI and spEPI: *KRT5*, *KRT15*, *KRT17*, *COL17A1*, *PDGFA* and *WNT3*), melanocytes (MEL: *PMEL*, *MLANA* and *FMN1*) and myocytes [MYO: *ACTC1*, *MYLPF* (*MYL11*), *TNNI1* and *MYH3*] (Fig. 2A-C, Fig. S1; see Table S1 for additional citations and notes for annotations) (Clay and Halloran, 2014; Ealy et al., 2016; Edqvist et al., 2015; Hartman et al., 2015; Kanton et al., 2019; Lahlou et al., 2018; Qu et al., 2016; Schneider et al., 2013; Wong et al., 2011). We validated the reproducibility of our results using another PSC line: the WA25 human ESC line. After processing and mapping of day 6 data from the WA25 with two distinct experiments using the WTC-DSP cell lines, we found that all cell clusters present in the DSP-GFP samples overlapped with cell clusters present in the WA25 samples, indicating that every cell type was present in each cell line prepared by each experimenter; however, the relative contribution did differ between cell types (Fig. S3A).

We used a combination of approaches to infer cell lineage trajectories, including RNA velocity analysis (scVelo; Fig. 2D), Monocle3-based trajectory analysis (Fig. S1C), the proximity of cell clusters across time (Fig. 2D, Fig. S1B), overlapping gene marker expression and *a priori* knowledge pulled from the developmental biology literature (Fig. 2D) (Groves and Fekete, 2012; Koehler et al., 2017). We started by manually tracing a trajectory of the otic lineage based on *a priori* knowledge and cell cluster proximity. We speculated that cells moved from the PSC1/2 populations, to *CLDN10^+^* surface ectoderm (cSE), to *PAX8/FGF8^+^* posterior pre-placodal ectoderm (pPPE), to *PAX8/PAX2^+^* otic-epibranchial progenitor domain cells (OEPD1/2), to *OC90/TBX2^+^* otic epithelium (OEP), to *OTOF/ATOH1^+^* hair cells (HC) and *NEUROG1^+^* neuroblasts (NEU1) (trajectory in Fig. 2D). However, we found that the RNA velocity and Monocle3 trajectory analysis failed to fully support this lineage progression (Fig. 2D, Fig. S1C). We suspected that the complexity of the integrated datasets may confound these trajectory inference approaches, so we split the datasets into two-timepoint pairs (Fig. S2). RNA velocity vectors were consistent across these paired down datasets (Fig. S2A), whereas Monocle3 trajectories were more biased by timepoint (Figs S1C, S2B); thus, we placed greater weight on RNA velocity vectors for inferring trajectories. In particular, the cell transitions among the surface ectoderm epithelia were complex. RNA velocity vectors within this region displayed a counterintuitive directionality away from the OEPD2 population towards early placodal (pPPE/aPPE/OEPD1) or epidermal (bEPI) progenitors (Fig. S2, days 3-10). However, the path to the OEPD2 cluster was unclear. In line with our expectations, vectors emanated from the OEPD2 cluster (and cycling SE cell cluster) toward the otic epithelial cluster (OEP) (Fig. S2, days 10-24). Yet the hair cell (HC) and the cluster containing presumptive otic neurons (NEU1) were disconnected from the OEP cluster. To reconcile these discrepancies and gain deeper insights, we analyzed the transcriptomic patterns during specific stages of inner ear organoid formation (days 0-3, 6-8, 10-13 and 18-36). In addition, we attempted to define the diversity of other cranio-pharyngeal epithelial, mesenchymal and neuro-glial cells emerging in the system (Fig. S1D).

### Days 0-3: emergence of neuroectodermal and surface ectoderm fates

We examined the transition from day 0 to day 3 to understand the founding populations of inner ear organoids. After 2 days of PSC aggregation in Essential 8 (E8) medium, we transferred the cell aggregates at day 0 to Essential 6 (E6) medium. Basal E6 medium with no additional components promotes anterior forebrain differentiation from pluripotent stem cells (Lippmann et al., 2014; Tchieu et al., 2017). We supplemented E6 with SB15432, a TGFβ inhibitor, to suppress mesendoderm induction and restrict differentiation to ectoderm cell types. We also added BMP4 to promote the induction of surface ectoderm (Chambers and Studer, 2011; Chambers et al., 2009; Koehler et al., 2013). Here, we selected a consensus BMP4 concentration (2.5 ng/ml) for otic induction; however, we have provided more in-depth BMP4 titration analyses in two of our related studies (Doda et al., 2023; van der Valk et al., 2023).

**Fig. 3.**
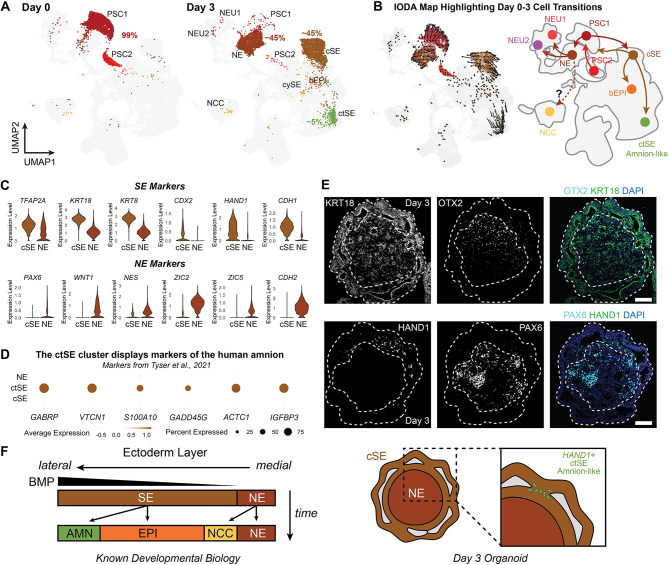
**Surface ectoderm and neuroectodermal cells emerging after 3 days of differentiation.** (A) UMAP plots of day 0 and day 3 inner ear organoids with cell type annotations show a clear development from pluripotent stem cells at day 0 to surface ectoderm and neuroectodermal cells at day 3. (B) Inferred development trajectories from day 0 to day 3 using RNA velocity analyses. (C) Marker gene expression of surface ectoderm (SE) genes and neuroectodermal (NE) genes at differentiation day 3. (D) Expression of amnion marker genes in the surface ectoderm clusters. (E) Representative images of day 3 inner ear organoids with surface ectoderm development in the outer layer (KRT18^+^) and neuroectodermal cells in the inner core (OTX2^+^ and PAX6^+^). HAND1^+^ cells are located within the outer surface ectodermal layer. Scale bars: 100 μm. (F) Schematic of known specification of the ectodermal germ layer *in vivo* and the presence of ectodermal lineages in the inner ear organoid, including amnion-like cells within the developing surface ectoderm.

To ensure proper differentiation, we confirmed by transcriptomic analysis that by day 3 and later, the genes associated with pluripotency, including *POU5F1* and *FOXH1*, are downregulated (Fig. S3B,C). The expression of BMP target genes *ID1*, *ID2*, *ID4*, *MSX2*, *BAMBI* and *BMPER* was upregulated in the cells collected on day 3, reflecting the treatment with BMP4 on day 0 (Fig. S4). The majority of cells at the day 3 time point can be divided into two groups: one group expressing surface ectoderm-associated markers, including *TFAP2A*, *KRT8*, *KRT18*, *CDX2*, *HAND1* and *CDH1* (clusters cSE and ctSE; hereafter, surface ectoderm (SE), ∼50% of the day 3 population) (Fig. 3A-C); another group expressing neuroectoderm-associated markers, such as *PAX6*, *WNT1*, *NES*, *ZIC2*, *ZIC5* and *CDH2* [hereafter, neuroectoderm (NE) cells, ∼45% of the day 3 population] (Fig. 3A-C). To compare the expression of our stem cell-derived organoid SE cells with an *in vivo* human correlate, we analyzed the expression of the SE markers observed in a gastrulating human embryo, including *GABRP*, *VTCN1*, *S100A10*, *GADD45G*, *ACTC1* and *IGFBP3* (Fig. 3D) (Tyser et al., 2021). This transcriptomic signature of the ctSE cluster matches with developing amnion cells (Tyser et al., 2021), and is further supported by recent amnion differentiation studies (Rostovskaya et al., 2022). To pinpoint the spatial locations of these cell populations, we performed immunohistochemistry (IHC) on day 3 organoids. The KRT18^+^ SE cells and the PAX6^+^/OTX2^+^ NE cells developed in the outer layer and inner core of the organoid, respectively (Fig. 3E). This outer layer of SE cells also contains moderate *POU5F1* expression, which could be confirmed by immunohistochemistry (Fig. S3B,C). We also noticed HAND1^+^ cells localized to a transitional cell population on the inner region of the outer SE layer (Fig. 3E,F). HAND1 expression by SE has been shown in the developing mouse and monolayer human stem cell differentiation and early ectoderm cells of the gastrulating human embryo, including amnion cells (Qiu et al., 2021 preprint; Tchieu et al., 2017; Tyser et al., 2021). These findings confirm that day 3 organoids have a ‘nested’ structure with surface ectoderm on the outer layer, neuroectoderm in the core and amnion-like cells in between.

### Days 6-8: FGF promotes the co-induction of posterior and anterior placodal ectoderm fates

We subsequently focused on the transition between day 6 and day 8 of differentiation. At these time points, many of the observed changes resulted from the day 3 treatment with FGF2 and LDN, a BMP inhibitor (Fig. 1A). These molecules mimicked the *in vivo* cues important for placode induction (Schlosser, 2006). Our transcriptomic analysis showed evident upregulation of the FGF target genes *SPRY1*, *SPRY2* and *DUSP6*, indicating the effect of FGF2 treatment (Fig. S4). Additionally, the expression of BMP target genes that were upregulated on day 3 was decreased by day 6, reflecting the treatment with the BMP inhibitor, LDN, on day 3 (Fig. S4).

**Fig. 4.**
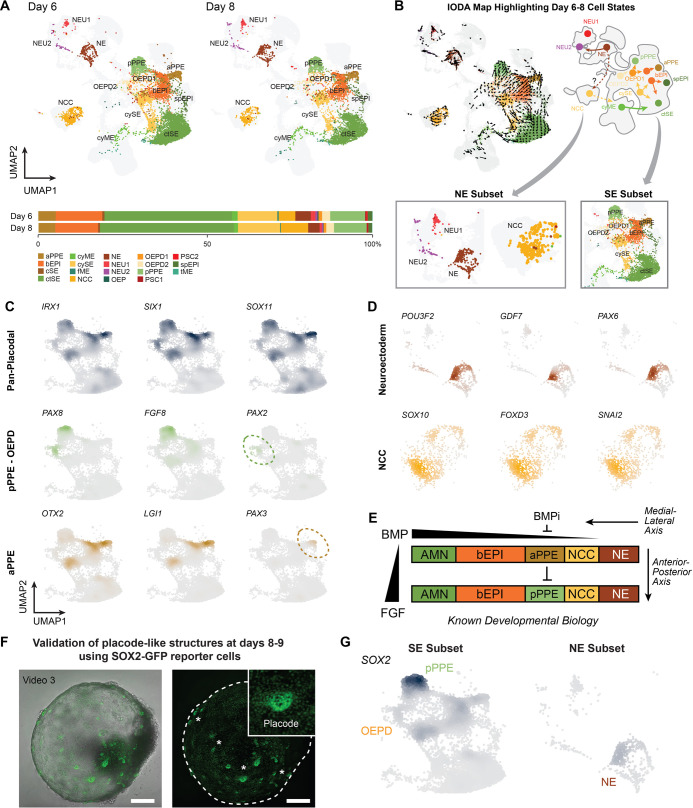
**Placode formation and neuroectoderm cell type specification during days 6 to day 8.** (A) UMAP plots of day 6 and day 8 inner ear organoids with cell type annotations show the development of both surface ectoderm and neuroectoderm into multiple cell types. Relative cell type proportion remains fairly constant between these two time points. (B) Inferred development trajectories at day 6 to day 8 calculated using RNA velocity and Monocle3 analysis. (C) Marker gene expression of pre-placodal ectoderm, separating pPPE with OEPD from aPPE. (D) Marker gene expression in neuroectoderm subsets (NE, NEU and NCC). (E) Known development of the ectoderm with segregation into specific ectodermal lineages, guided by gradients of BMP and FGF. (F) SOX2-GFP expression in a WTC reporter line, highlighting SOX2-GFP in placode-like structures between days 8 and 9. Scale bars: 250 μm. See also Movie 3. (G) Density plots depicting *SOX2* expression in SE and NE subsets.

FGF2 treatment on day 3 induced differentiation of the outer layer of the SE toward a placode fate. However, the efficiency of anterior versus posterior pre-placode induction was unclear. We sought to investigate whether multiple subtypes of placode tissue were induced. Looking first at presumptive derivatives of the day 3 surface ectoderm population, we identified two distinct subtypes of cranial pre-placode tissue, consisting of posterior pre-placodal ectoderm (pPPE, 10.1% of the day 6-8 aggregate) and anterior pre-placodal ectoderm (aPPE, 5.1%), as well as OEPD1 and OEPD2 (3.3%, Fig. 4A). We first focus on the surface ectoderm (SE) subset (clusters cySE, ctSE, spEPI, bEPI, aPPE, pPPE, OEPD1 and OEPD2), to gain a more detailed view of the placode populations from days 6 and 8 (Fig. 4B). Within this subset, we observed the expression levels of *IRX1*, *SIX1* and *SOX11*, confirming broad cranial placode identity (Fig. 4C, Fig. S5A) (Riddiford and Schlosser, 2016; Schlosser and Ahrens, 2004; Sullivan et al., 2019). Within the identified subset of placode cells, we found a region that expressed *OTX2*, a marker of aPPE development (Fig. 4C, Fig. S6A) (Steventon et al., 2012). Additionally, some of those aPPE cells expressed *LGI1*, a gene expressed in anterior surface ectoderm and olfactory placodes (Silva et al., 2011), and *PAX3*, a marker of trigeminal placode development (Baker et al., 2002; Logan et al., 1998; Riddiford and Schlosser, 2016; Tchieu et al., 2017) (Fig. 4C). Although the trigeminal placode has not always been considered an anterior placode, its development requires *OTX2*, a known anterior placodal marker (Steventon et al., 2012). We also discerned a cell cluster separate from the aPPE cells and consistent with the pPPE, the tissue from which the otic and epibranchial placodes derive. The pPPE was identified by *PAX8, FGF8* and *GBX2* expression (Fig. 4C, Fig. S6A). *FGF8* is an important molecule for otic placode development expressed in the endoderm underlying the otic placode and the developing otic placode itself (Adamska et al., 2001; Ladher et al., 2005; Léger and Brand, 2002). Considering the role FGF signaling plays in inducing the otic placode from pPPE, this highlighted the likely importance of endogenous signaling throughout organoid induction (Martin and Groves, 2006; Wright and Mansour, 2003; Wright et al., 2003; Yang et al., 2013) (Fig. 4E). We also identified a cluster of OEPD2 and, to a lesser extent, OEPD1 cells expressing both *PAX8* and *PAX2* (Freter et al., 2012).

Upon further investigation of the genes expressed by pPPE cells, surprisingly, we identified *NKX2-5* as a specific marker, a master regulator of cardiac development (Fig. S6B) (Tanaka et al., 1999; Zhang et al., 2014)*.* Previous literature has indicated that the lateral-most epithelia in the pharyngeal region of the developing mouse embryo express NKX2-5 (Fig. S6C) (Hidaka et al., 2010; Moses et al., 2001; Tanaka et al., 2001). NKX2-5 is also expressed in the developing heart mesoderm in the mouse, which underlies the developing pPPE (Litsiou et al., 2005). We further confirmed NKX2-5 protein expression in organoid epithelial cells at day 6 and day 8 by immunohistochemistry (Fig. S6D-E). The presence and location of these NKX2-5^+^ cells suggested that the organoid system recapitulates a broad medial-to-lateral range of developing epithelia in the pharyngeal region.

Based on previous results, one of our central assumptions about germ layer induction in the early stages of inner ear organoid differentiation was that TGFβ inhibition on day 0 prohibits mesoderm induction (Koehler et al., 2017). To assess mesoderm development in our datasets, we investigated the expression of *TBXT*, a marker of mesoderm development, and found it absent (Rivera-Pérez and Magnuson, 2005; Smith, 1999; Wilkinson et al., 1990). We also performed immunohistochemistry on day 6 and found no expression of brachyury, the protein product of *TBXT* (data not shown). Likewise, we probed the dataset for *SOX17*, a representative marker of endoderm development. We found its expression in fewer than 20 cells scattered throughout the clusters, supporting our initial assertions that mesendoderm is lacking in these organoid cultures (Séguin et al., 2008; Wang et al., 2011; Yu et al., 2021).

Next, we focused on the day 6 to day 8 neuroectoderm-derived populations (Fig. 4B), which could be subdivided into neural crest expressing (*SOX10*, *FOXD3* and *SNAI2*), neuroectoderm expressing (*POU3F2*, *GDF7* and *PAX6*) as well as early neurons (Fig. 4D). The shared expression of FOXD3 between the NE and NCC clusters suggests a potential developmental relationship, indicating that NCC cells in our organoid system stem from a neural ectodermal origin (Fig. S5B). This NCC cluster likely serves as the progenitor pool for Schwann cells detected in late-stage inner ear organoids, as evidenced by their expression of early glial markers (Fig. S5C) (Tasdemir-Yilmaz et al., 2021; van der Valk et al., 2023). Additionally, we concluded that the NCC generated in this culture system is generally of a cranial identity due to the presence of chondrocyte development, as the cranial NCC is the only type of NCC capable of chondrogenesis (Mori-Akiyama et al., 2003; Simoes-Costa and Bronner, 2016; Takahashi et al., 2001; Vaglia and Hall, 1999). This cranial NCC identity is additionally supported by the very low expression of HOX genes (Fig. S7) (Creuzet et al., 2002).

The development of the surface ectodermal as well as neuroectodermal cell lineages correspond with the segregation of developing ectoderm, during which both BMP and FGF gradients are essential for anterior-to-posterior and medial-to-lateral cell type diversity (Fig. 4E). Using the WTC hiPSC cell line containing a fluorescent reporter for SOX2 [SOX2-mEGFP (SOX2-GFP)], we can visualize SOX2 expression in the emerging pPPE, OEPD1, OEPD2 and NE structures at days 8-9 (Fig. 4F,G, Movie 3). These data suggest that the divergent ectodermal lineages that arise during organoid generation form a mixed population of surface ectoderm, neuroectoderm and pharyngeal tissue. Overall, at these day 6 and 8 time points, our data suggest that FGF and LDN treatment on day 3 induces SE tissue to generate a broader diversity of cranial placode tissue then we originally appreciated (Fig. S5D).

### Days 10-13: GSK3β inhibition primes organoids for otic specification

**Fig. 5.**
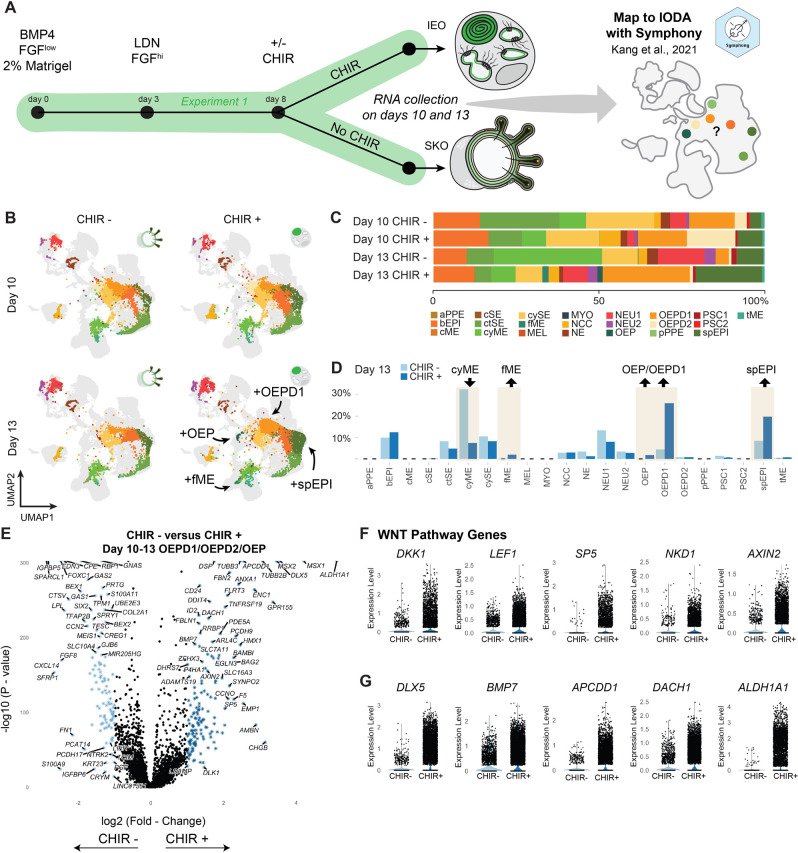
**The role of CHIR treatment in inner ear organoid induction.** (A) Overview schematic of CHIR treatment and RNA sample collection days. Mapping of the data was performed using Symphony, an R package for reference atlas mapping. (B) UMAPs produced from the reference atlas mapping for both time points (day 10 and day 13) and both conditions (‘CHIR−’ for those treated without CHIR and ‘CHIR+’ for those treated with CHIR). (C) Relative cell type contribution per time point and condition. (D) Percentage of cell types per condition at day 13, with increased OEPD1 and OEP cells in the CHIR-treated condition. (E) Volcano plot depicting differentially expressed genes within the OEPD1, OEPD2 and OEP clusters on day 10 and day 13 between non-treated and CHIR-treated organoids. (F) Violin plots showing expression of WNT pathway gene expression within the OEPD and OEP clusters on day 10 and day 13 between non-treated and CHIR-treated organoids. (G) Gene expression of some differentially expressed genes within the OEPD1, OEPD2 and OEP clusters on day 10 and day 13 between non-treated and CHIR-treated organoids.

A pivotal event during inner ear organoid induction is treatment with the GSK3β inhibitor CHIR-99021 (CHIR), starting on day 8 and continuing until day 15. We focused our analysis to the day 10-13 time points to investigate the effects of CHIR treatment and WNT pathway activation on otic vesicle induction (Fig. 5A). Inhibiting GSK3β activates the WNT signaling pathway, a molecular pathway implicated in directing cranial placode tissue towards an otic fate (Ohyama et al., 2006). Without the addition of CHIR, otic placodes fail to form and the developmental trajectory of the organoid epithelium becomes biased to a hair-bearing epidermal fate (DeJonge et al., 2016; Koehler et al., 2013, 2017; Lee et al., 2020). Here, we examined the effect of CHIR on cell type induction at single-cell resolution by comparing day 10 and day 13 datasets derived with or without CHIR treatment obtained during Experiment 1 (Fig. 5A). We mapped the datasets from these four conditions (days 10 and 13, CHIR-treated and control) to the IODA using a package called Symphony (Fig. 5A,B) (Kang et al., 2021). The IODA overview UMAP already contained day 10 and 13 CHIR-treated data (Fig. 2A); however, we chose to remap these data and look for deviations in order to validate the Symphony workflow.

After mapping each condition, we determined the proportional contribution of each cell type (Fig. 5C). The day 10 and 13 CHIR-treated cells overlapped with the identical data points found in the overview UMAP (Fig. 1A), giving us confidence in the Symphony approach. CHIR treatments result in a clear increase of the OEPD1 and OEP cell types, as well as in suprabasel epidermal cells (spEPI) and *FOXC1^+^* mesenchymal cells (fME), at day 13 (Fig. 5C,D). Although OEP cells appear to be linked to the OEPD2 cells, the significance of OEPD1 cells is less clear. Interestingly, based on RNA velocity, OEPD1 cells appear to be primarily oriented toward epidermal progenitors (bEPIs) and not towards OEPD2 or OEP induction (Fig. 2D and Fig. S2, days 10-13). To assess the effect of CHIR treatment on the OEPD1, OEPD2 and OEP cluster on day 10 and day 13, we show differentially expressed genes between non-treated and CHIR-treated conditions (Fig. 5E). When specifically analyzing WNT target genes, we see upregulation in the CHIR-treated conditions (Fig. 5F). This suggests that the WNT pathway was indeed activated in the desired cells.

To analyze signaling factors that influence the developing otic tissues after CHIR treatment, we compared the differential expression of genes in the OEPD1 and OEPD2 cells on day 10 and day 13 with and without CHIR treatment. We found that the expression levels of several genes were upregulated in the CHIR-treated OEPD1, OEPD2 and OEP cells, including *BMP7*, *DLX5*, *APCDD1* and *DACH1* linked to inner ear development and function (Ayres et al., 2001; Groves and Bronner-Fraser, 2000; Jukkola et al., 2004; Miwa et al., 2019); *ALDH1A1*, which encodes the retinoic acid synthesizing enzyme, was one of the most upregulated, particularly in the OEPD1 cluster (Fig. 5G). Similarly, *ALDH1A1* expression upregulation occurred after CHIR treatment in directed differentiation systems for other cell types, such as midbrain dopaminergic neurons (Kim et al., 2021). Additionally, in cancer cell signaling, β-catenin, a WNT pathway intermediary, plays an important role in *ALDH1A1* expression regulation (Cojoc et al., 2015; Condello et al., 2015). *ALDH1A1* expression upregulation may therefore be a cellular response that is specific to CHIR treatment. We also noted that TGF signaling (*MSX1*, *MSX2*, *ID1*, *ID2* and *ID4*), Hedgehog signaling (*HHIP*) and Notch signaling (*NRARP*, *HES1* and *NOTCH1*) mediators are upregulated after CHIR treatment (Table S1). Together, we confirm WNT activation after CHIR treatment and the induction of OEP cells with an increased otic transcriptomic signature; however, our results also suggest that CHIR treatment is supportive of epidermal induction.

### Days 18-36: diversification and terminal differentiation of mesenchymal, neuronal and otic cell types

Our analysis of the later time points (days 18-36) focused primarily on the inner ear cell types. These later stages of maturation took place mainly via self-organization without exogenous morphogenic signaling factors. We generated subsets specific to the cell type of interest to focus the analysis of these later time points.

#### Identification of inner ear-related periotic mesenchyme

By analyzing the connective tissue surrounding the inner ear organoids, we identified a population of POU3F4^+^ cells close to the epithelia and developing neurons in day 36 organoids (Fig. S8A). These cells, also expressing *OTOR*, are a subtype of the *FOXC1*^+^ fME cluster (Fig. S8B) (Wilkerson et al., 2021). These periotic mesenchymal-like cells are essential for proper development (Huang et al., 2020) and function of the inner ear, as reviewed by Furness (2019).

#### Inner ear organoid induction yields diverse neuronal populations

**Fig. 6.**
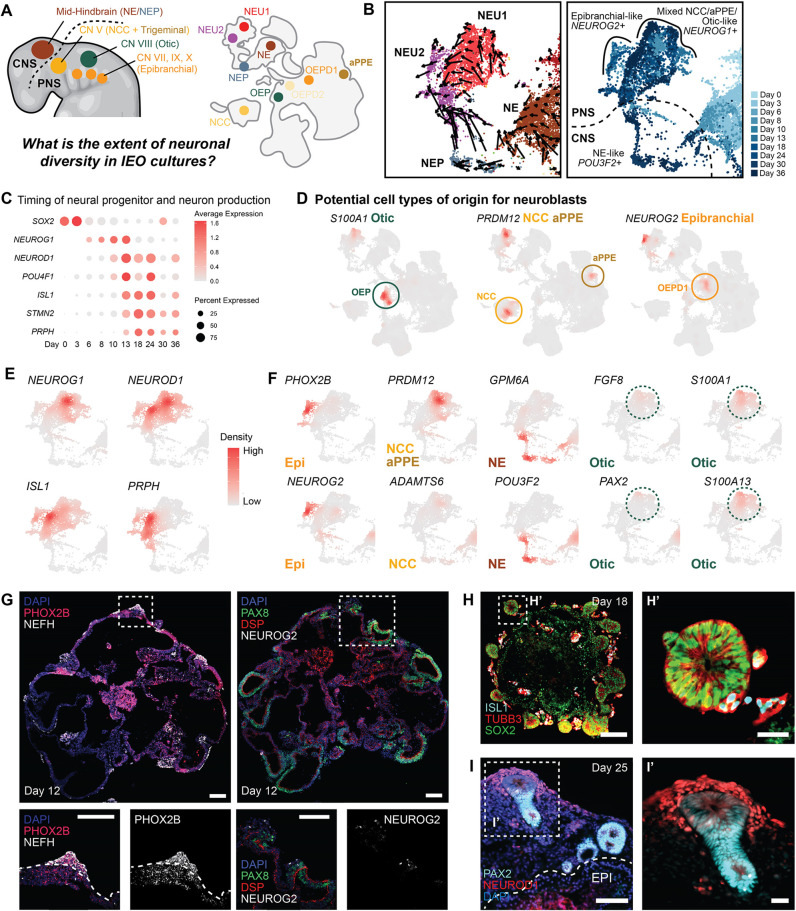
**Neuronal diversity during inner ear organoid differentiation.** (A) Overview of the cranial neuronal diversity seen in the developing embryo, which is partly captured in the inner ear organoid system. (B) RNA velocity and temporal analyses of immature neuroblast and neuroepithelial populations that give rise to more mature neurons. (C) Marker gene expression of immature and more-mature neuronal genes in the NE, NEU1, NEU2 and NEP clusters at different time points. (D) Identification of the four neuron populations: otic-like, neural crest cell-like (NCC), anterior pre-placodal-like (aPPE) and epibranchial-like. (E) Gene marker expression throughout the neuronal population showing more immature (*NEUROG1* and *NEUROD1*) and mature (*ISL1* and *PRPH*) genes. (F) Different populations of neurons identified by marker gene expression. (G) Epibranchial neurons in inner ear organoids. Scale bars: 100 μm. (H) Putative ISL1^+^ delaminating neuroblasts in close proximity to SOX2^+^ otic vesicles in a day 18 organoid. Scale bars: 100 μm in H; 25 µm in H′. (I) NEUROD1^+^ precursor neurons surrounding PAX2^+^ otic vesicles in a day 25 organoid. Scale bars: 100 μm in I; 25 µm in I′. CN, cranial nerve; EPI, epibranchial.

Within the full integrated dataset, we identified neuronal cell clusters, confirmed by the expression of *NEFM*, *STMN2*, *TUBB3* and *ANKFN1* (Figs 2E and 6). Within the NEP, NEU1 and NEU2 clusters, we observed a clear gradient of immature (*NEUROG1*^+^ cells) to mature (*STMN2*^+^ and *PRPH*^+^ cells) subtypes (Fig. 6C,E). Within the subset, we identified cells with identities corresponding to four separate neuron-generating populations: epibranchial placode (*PHOX2B* and *NEUROG2*), neural crest (*PRDM12* and *ADAMTS6*), neuroectoderm (*GPM6A* and *POU3F2*) and otic vesicle (*FGF8*, *S100A1*, *PAX2* and *S100A13*) (Fig. 5D-F) (Fode et al., 1998; Grillet et al., 2003; Kupari et al., 2019). In addition to the otic vesicle-like neuronal population, we observed a more immature otic neuroblast population, marked by expression of *FGF8* and *PAX2* (Fig. 5F) (Appler and Goodrich, 2011; Appler et al., 2013; Li et al., 2020; Lu et al., 2011; Sandell et al., 2014; Yu et al., 2013). There was a clear order to the generation of these neurons. The development of epibranchial placode-like and NCC/aPPE neurons was observed in earlier datasets, whereas the otic neurons were observed later.

As we previously focused on characterizing otic neurons by immunohistochemistry (Koehler et al., 2017), here we looked more specifically at the developing epibranchial placode. We stained day 12 samples for *PHOX2B* and *NEUROG2*. *NEUROG2* is a determination factor for epibranchial-derived neurons (Fode et al., 1998), and *PHOX2B* is a marker employed by Kupari et al. to distinguish epibranchial neurons from neural crest-derived neurons within the mouse vagal sensory ganglia (Kupari et al., 2019). To confirm the pPPE origin of the epibranchial placode, we also stained for *PAX8*. Our immunohistochemistry analysis showed regions of PAX8^+^/DSP^+^ pPPE giving rise to PHOX2B^+^/NEFH^+^ cells (Fig. 6G). Some of these cells also expressed NEUROG2 (Fig. 6G), confirming the presence of epibranchial neurogenesis in inner ear organoid culture. In addition, ISL1^+^, TUBB3^+^ and NEUROD1^+^ neurons are present in close proximity to the developing otic vesicles at day 18 to day 25 (Fig. 6H,I). Overall, our data reveal a broader diversity of neuronal subtypes than we had previously appreciated in this organoid system.

#### Maturation and diversification of the otic epithelium

**Fig. 7.**
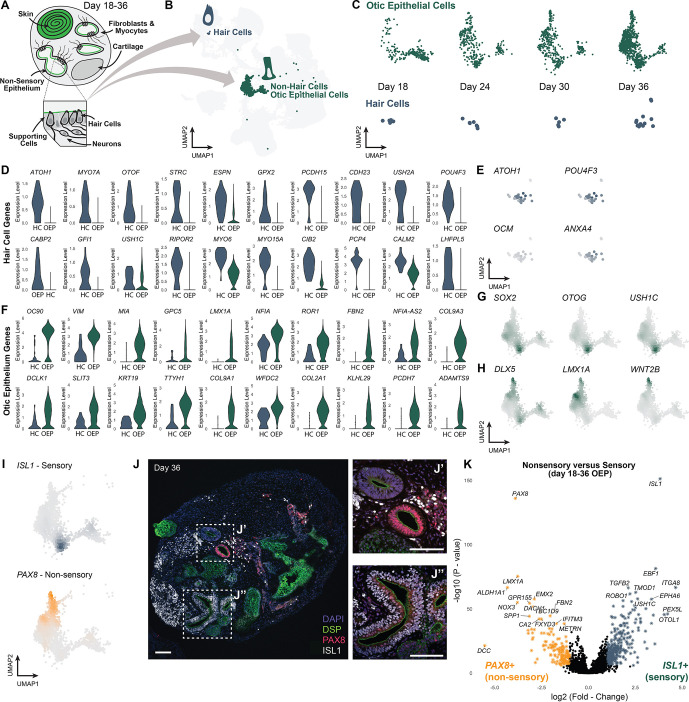
**Otic epithelium and hair cells between day 18 and day 36 of differentiation.** (A) Illustration of the cellular diversity in the inner ear organoid, containing otic epithelium and hair cells located in vesicles within a later-stage organoid. (B) The otic epithelium (OEP) and hair cell (HC) clusters in the Inner Ear Organoid Developmental Atlas (IODA). (C) Both OEP and HC clusters show an increase in cells over time. (D) The transcriptomic analysis of hair cell markers indicates a certain level of maturity. (E) Feature plots of hair cell (HC) transcription factors (*ATOH1* and *POU4F3*) as well as type I (*OCM*) and type II (*ANXA4*) vestibular hair cell marker genes. (F) The otic epithelial cells expressed definitive markers confirming otic epithelium identity. (G,H) Density plots displaying distinct expressions of sensory markers (*SOX2*, *OTOG* and *USH1C*) and nonsensory markers (*DLX5*, *LMX1A* and *WNT2B*) visible in the OEP cluster. (I) Sensory versus nonsensory identity can be distinguished based on *ISL1* and *PAX8* expression, respectively. (J) Within the day 36 organoid, some vesicles showed expression of PAX8 (J′, nonsensory) and some showed expression of ISL1 (J″, sensory), but rarely displayed dual expression of both proteins. Scale bars: 50 µm. (K) Differentially expressed genes between nonsensory (*PAX8* expressing) and sensory (*ISL1* expressing) day 18 to day 36 OEP cells.

The nascent otic vesicle epithelium in the embryo is patterned by local and self-generated signaling cues to produce an intricate labyrinth of sensory (i.e. containing supporting cells and hair cells) and nonsensory epithelia. To investigate the developing otic epithelium in organoids, we extracted a subset of cells from the IODA overview plot containing putative otic epithelial cells and hair cells (Fig. 7A-C). Focusing on days 18-36, we stratified these two clusters by day and, surprisingly, observed cells with a hair cell-like transcriptional signature as early as day 18, with increasing numbers through day 36 (Fig. 7C). Despite the early hair cell transcriptional profiles, we had difficulty identifying MYO7A^+^ hair cells by immunohistochemistry before day 30 of differentiation (Fig. 1D). Transcriptional upregulation of hair cell-associated genes may occur well before the cells express detectable levels of hair cell-associated proteins. Next, given the unique gene expression program of native hair cells in mice and humans, we sought to characterize the transcriptional identity of human organoid-derived hair cells. Unbiased marker analysis showed that the hair cell cluster expressed numerous genes associated with hair cell identity. We noted the expression of key transcriptional regulators of hair cell fate, such as *ATOH1*, *GFI1* and *POU4F3*, as well as genes encoding crucial components of the hair bundle, such as *MYO7A*, *STRC*, *ESPN*, *GPX2*, *PCDH15*, *CDH23*, *USH2A*, CABP2, *USH1C*, *RIPOR2*, *MYO6*, *MYO15A*, *CIB2*, *PCP4*, *CALM2*, *LHFPL5* and *OTOF*, a gene encoding a calcium-sensing protein in hair cells (Fig. 7D) (Burns et al., 2015; Cai et al., 2015; Diaz-Horta et al., 2014; Jones et al., 2006; Kolla et al., 2020; Ku et al., 2014; Liu et al., 2014; Scheffer et al., 2015; Wilkerson et al., 2021; Xiang et al., 1997). Day 36 remains an early time point for hair cell induction (roughly equivalent to fetal weeks 7-8 in the fetus); thus, unsurprisingly, we were unable to resolve clear vestibular or cochlear hair cell identities based on known markers (Burns et al., 2015; Kolla et al., 2020; Wilkerson et al., 2021). Consistent with the results of our previous study (Koehler et al., 2017), the hair cell expression profiles most closely mirrored that of *ANXA4*^+^ type II vestibular-type hair cells, whereas there was limited expression of the type I vestibular hair cell marker gene, oncomodulin (*OCM*) (Fig. 7E). ANXA4 has also been described to be expressed in more immature vestibular hair cells (McInturff et al., 2018). In addition, we see that from the first transcriptional emergence of the hair cell population at day 18, these hair cells undergo maturation up to day 36 based on their gene expression, with loss of SOX2 and ATOH1, and acquisition of more late stage hair cell markers (Fig. S9A).

Finally, we focused on better defining the otic epithelial cells. The otic epithelium expressed markers specific for the otic placode, such as *PAX8*, *PAX2*, *SIX1*, *EYA1* and *FOXG1* and more mature otic epithelium, such as *OC90*, *HMX3*, *DLX3*, *SALL4*, *DUSP6*, *SPRY2*, *LMX1A*, *OTOA*, *APOE*, *SMOC2*, *SPARCL1*, *FBXO2*, *COL11A2* and *COL9A2* (Durruthy-Durruthy et al., 2014; Ealy et al., 2016; Groves and Bronner-Fraser, 2000; Hartman et al., 2015) (Fig. 7F). Notably, the transcription factor *TBX2* was highly differentially expressed in organoid otic epithelial cells and was recently shown to be a key transcriptional regulator of otic fate specification (Fig. S9B) (Hu et al., 2006; Kaiser et al., 2021). We performed *in situ* hybridization to confirm *TBX2* transcripts, specifically in inner ear organoids (Fig. S9C). We have previously seen some signatures reminiscent of developing cochlear duct cells (van der Valk et al., 2023); in line with this, we see that both markers for vestibular and cochlear epithelium are expressed in the OEP (Fig. S9D) (van der Valk et al., 2023; Wilkerson et al., 2021). Investigating the development of specifically cochlear cell types using this organoid system is an ongoing pursuit. Next, we wondered whether the organoid epithelial cell cluster could be subdivided into sensory and nonsensory epithelial cells. Unbiased clustering revealed a distinct region of the otic epithelial cluster marked by sensory genes, such as *SOX2*, *OTOG* and *USH1C* (Fig. 7G). In general, sensory-like cells clustered away from cells expressing nonsensory markers, such as *DLX5*, *LMX1A* and *WNT2B* (Fig. 7H) (Li et al., 2004; Radde-Gallwitz et al., 2004). *ISL1* and *PAX8* could be used to distinguish between the sensory and nonsensory populations, respectively (Fig. 7I and Fig. S9E). Interestingly, when we performed immunohistochemistry on day 36 aggregates, we found that inner ear epithelium expressed either ISL1 (sensory) or PAX8 (nonsensory), and rarely displayed their co-expression (Fig. 7J, Fig. S9F,G). Differentially expressed genes between these two epithelial types (Fig. 7K) reveal expression of *ALDH1A1* and *LMX1A* in the nonsensory epithelial cells, and *ITGA8*, *EBF1* and *USH1C* in sensory epithelia (Buckiová and Syka, 2009; Evans and Müller, 2000; Hatch et al., 2007; Kagoshima et al., 2023 preprint; Miwa et al., 2019; Nichols et al., 2008; Romand et al., 2004; Simmler et al., 2000; Tarchini et al., 2018; Tian et al., 2010), confirming the presence of both types in our organoid model. Together, our findings reveal distinct patterning of organoids undergoing maturation. Future studies could investigate the signaling mechanisms controlling epithelial cell fate and attempt to bias cultures toward sensory or nonsensory fates, depending on the biological or clinical question.

## DISCUSSION

**Fig. 8.**
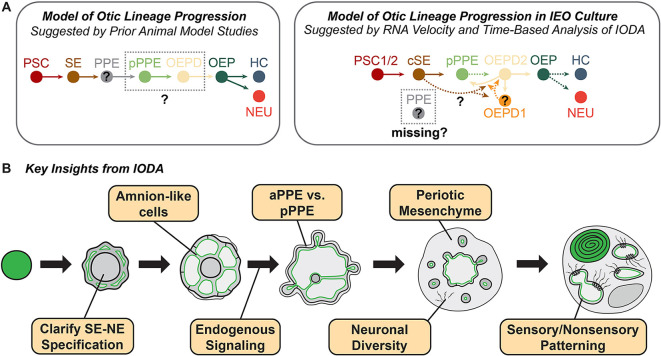
**Insights provided by the Inner Ear Organoid Developmental Atlas.** (A) Comparison of otic lineage progression models based on the literature and the Inner Ear Organoid Developmental Atlas (IODA) data. Dotted arrows represent speculative connections. In animal models, it remains uncertain whether a generic pre-placodal progenitor cell state exists (indicated as PPE in gray) or whether there are distinct ‘pPPE’ or ‘OEPD’ cell states (gray outline with a question mark). The IODA data do not display a distinct PPE progenitor, only cells expressing either posterior or anterior placode markers. *PAX8/PAX2^+^* OEPD2 cells appear to produce OEP cells, as expected, yet ambiguity remains regarding the origin of OEPD2 clusters, their interplay with other posterior placodal cells in the dataset and the *in vivo* relevance of cells in the largely CHIR-induced OEPD1 cluster. (B) Schematic showcasing previously unreported features of inner ear organoid generation, as revealed by the IODA.

In this study, we used single-cell transcriptomics to chart the development of inner ear organoids over 1 month in culture. Our data show that, like *in vivo* development inner ear tissue in the organoid model forms in a diverse environment of cranial mesenchymal and ectodermal cell types, and uncovers complex cell state transitions that will require additional work to fully resolve (Fig. 8A) (Baker and Bronner-Fraser, 2001). In addition to clarifying the development of the otic lineage, the IODA confirms the identity of many known and previously unreported induced cell types, such as neural crest cells, anterior pre-placode, epibranchial neurons and neuroectodermal cells. Our findings provide valuable insights into the cellular and molecular mechanisms underlying oto-pharyngeal development, and shed light on the development of additional cell types that are crucial for proper inner ear formation (Fig. 8B). We envision the IODA as a benchmark with which future datasets from organoids or human fetal/adult inner ear specimens can be compared, much like reference atlases under development for other organ systems (Haniffa et al., 2021; Yu et al., 2021). Since this study was posted as a preprint in 2021, additional work has emerged profiling inner ear organoid cells at various stages of differentiation and with different signaling treatments (Doda et al., 2023; Moore et al., 2023; Nie et al., 2022; Ueda et al., 2023; van der Valk et al., 2023). Data from these and future works can be easily integrated with or mapped to the IODA to gain additional insights with packages such as Harmony and Symphony.

Our analysis shows that inner ear organoids transit through specific developmental stages beginning with an inside-out patterning of neural-to-surface ectoderm. The nested structure of organoids at day 3 reflects the medial-to-lateral organization of the ectoderm germ layer during neurulation, including putative extra-embryonic amnion cells (Rostovskaya et al., 2022). Likewise, the day 3 organoids mimic the concentric ring structure found in 2D hPSC colonies grown on micropatterned plates in the presence of SB and BMP4 (Etoc et al., 2016). The initial establishment of surface ectoderm is a foundation event for successful otic induction, and likely involves an interplay of chemical (e.g. BMP) and mechanical cues. Thus, the IODA data can provide a starting point for a deeper investigation of the mechanisms controlling neural-to-surface ectoderm patterning, focusing on starting aggregate size, matrix composition and additional chemical signaling cues.

The emergence of mesenchymal cells in inner ear organoid cultures remains unclear and will require follow-up analysis (see Fig. S1D for areas of interest). Later in the culture, we identified multiple progenitor populations clustered next to, and potentially giving rise to, mesenchymal-like cells, such as *TWIST2^+^* fibroblast-like and *MATN1+* chondrocyte-like cells (the tME and fME clusters). Our data here and in a previous study (Lee et al., 2020) suggest that neural crest progenitor cells contribute to mesenchymal cell populations; however, cranial or pharyngeal mesoderm-like cells may also emerge in these cultures. Although the NCC and cyME populations are disconnected, we note *TWIST1* expression in the NCC population (see supplementary Materials and Methods QC analysis section). Additionally, the development of myocytes (MYO) and their close association with OEPD2 cells suggests a non-NCC source of mesenchyme. Interestingly, these myocytes have been identified as cranial skeletal myocytes in a separate study looking at later-stage inner ear organoids (van der Valk et al., 2023). Typically, skeletal myocytes are derived from the paraxial or lateral plate mesoderms; however, our model does not appear to induce typical *TBXT^+^* mesodermal progenitors. In addition, in our later-stage organoids, we have also identified endothelial cells, which are atypical derivatives of the neural crest (van der Valk et al., 2023). This raises the question of what the alternative source for these cell types might be. Potentially anterior somitic mesoderm or cranial mesoderm, both of which are poorly characterized mesodermal subtypes. A recent study by the Marioni lab found that anterior somitic mesoderm arises in the absence of *TBXT* expression and shows expression of *HAND1*, *PRRX1* and *PRRX2* genes similar to the lateral plate mesoderm and the cyME, fME and tME populations in our organoid data (Guibentif et al., 2021). Notably, the somite marker *MEOX1* is expressed in the OEPD2 cluster adjacent to the MYO cluster (Table S1). Somites are transient epithelial structures that undergo epithelial-to-mesenchymal transition to give rise to various muscles, such as those found in the tongue and neck (Frisdal and Trainor, 2014). Anterior somites develop in close proximity to the developing pre-placodal and OEPD region. However, it is difficult to draw any firm conclusions from the limited markers in our organoid system. Finally, the cME population emerges in day 18-36 datasets and expresses several otic epithelial markers (*USH1C* and *OTOG*), as well as mesenchymal markers (*CRYM* and *LUM*). Based on RNA velocity analysis (Fig. 2 and Fig. S2), the cME may arise from OEP cells, which would be an unexpected and potentially unique feature of the organoid model. We suspect lineage tracing will be needed to fully clarify which progenitor cell populations contribute to the emerging mesenchyme in IEO cultures. Notably, convergent cell lineage commitment has been well documented in the literature (Wagner and Klein, 2020). Single-cell time course analysis of developing zebrafish revealed a similar phenomenon of common mesenchymal cell types emerging from cranial neural crest cells, pharyngeal mesoderm cells and somites (Wagner et al., 2018). Unfortunately, reference data for cranial and pharyngeal mesoderm cell populations is lacking. It is unclear whether the cME, tME and fME cells represent a distinct cell type found in mouse or human embryos. A deeper understanding of mesenchyme lineages could help guide efforts to precisely engineer organoids with various periotic mesenchymal cells, such as spiral limbus fibrocytes, a cell population implicated in certain forms of congenital hearing loss (Furness, 2019).

The mechanistic insights from the IODA should aid researchers in generating inner ear organoids with greater efficiency and fidelity. Our initial analysis points to several key cell fate decisions that could be adjusted to improve on-target development. In particular, the induction of posterior pre-placodal ectoderm (pPPE) and OEPD induction was surprisingly inefficient. More-efficient generation of the pPPE/OEPD at the expense of aPPE would increase the abundance of tissues responsible for otic vesicle generation. Our data suggest several endogenously produced FGFs may play a role in this process. Future work could re-evaluate the use and concentration of FGF2 applied on day 3 of our current culture. The newly identified amnion-like epithelial cell population (ctSE) should be a crucial target for improving on-target otic specification. Our analysis shows that Notch signaling is engaged in this cell population. In addition, our analysis confirms a vestibular type identity of inner ear organoids and could be leveraged to work toward generating cochlear organoids through modulation of sonic hedgehog, WNT and BMP signaling (Moore et al., 2023; Nist-Lund et al., 2022). Previous studies have shown that WNT activation patterns the otic vesicle towards more dorsal (vestibular) cell fates (Chatterjee et al., 2010). Although CHIR treatment is necessary for otic induction, it is clear from our data that the global level of WNT-related gene expression remains elevated from the time of CHIR treatment onwards (days 8-36; Fig. S3). As recent work suggests, fine-tuning the activation and inhibition of WNT could be crucial for deriving regions of the otic vesicle harboring cochlear duct progenitors (Moore et al., 2023) and should continue to be the focus of future research.

Our study provides a resource for further investigating the development of hair cells and other craniofacial tissue types in organoid cultures. The ability to grow hair cells *in vitro* is a fascinating prospect, given that mammalian hair cells are susceptible to irreversible degeneration, and human samples are scarce (Bermingham-McDonogh et al., 2012). Our time-based analysis provides a framework for developing cell or gene therapies for hearing loss and imbalance.

### Limitations of the study

There are several limitations to our experimental design. First, our single-cell data are limited to a time series collected from one cell line. Using additional cell lines for comparison would strengthen our conclusions and provide insight into the consistency of otic induction across cell lines. We compared WA25 hESCs and DSP-GFP cells on day 6 of differentiation and observed consistent cell populations across both lines. Furthermore, recent work by our groups evaluated the differentiation efficiency over multiple PSC lines and validated robust induction of later-stage inner ear organoids, including analyses at the single-cell level (van der Valk et al., 2023). Another limitation is that we collected cells from two separate experiments (days 0-13 and 18-36). One continuous experiment or a comparison of the same time points across multiple experiments would provide a clearer picture of organoid heterogeneity – new fixed-sample protocols could make these experiments easier to execute. Given the complex diversity of cell types we have identified here, we expect there are likely minority cell populations not captured in the 6000-10,000 cells per time point or cells that evaded detection in our analysis. This investigation is an initial step toward a reference atlas of human inner ear organoids and human otic development in general; however, another key limitation is the lack of stage-matched human fetal inner ear reference data for comparison (Doda et al., 2023). Last, unexpected or strange features of the cell lineages inferred from UMAP plots, could be partially attributed to the limitations of relying on this method of dimensionality reduction, as recently discussed by Chari and Pachter (Chari and Pachter, 2023). Our related studies comparing organoids to human fetal and adult inner ear tissue overcome many limitations but do not examine the early inductive events shown here (Doda et al., 2023; van der Valk et al., 2023). Future datasets, in organoids and fetal specimens, using approaches that provide more transcriptomic information (e.g. Smart-Seq or VASA-seq) or more insight into chromatin state (e.g. ATAC-seq, SHARE-seq) will be needed to further resolve inner ear organogenesis at single-cell resolution (Ma et al., 2020). We encourage readers to dig deeper into our dataset to make additional discoveries.

## MATERIALS AND METHODS

No statistical methods were used to predetermine sample size. The experiments were not randomized, and the samples were not anonymized to investigators during experiments and outcome assessments.

### hPSC culture

Stem cell differentiation experiments were performed primarily with the wild-type C (WTC) human iPSC line (passage 35-51) obtained from the Allen Institute for Cell Science and the Coriell Institute (Miyaoka et al., 2014). Within the Allen Institute's catalog of fluorescent reporter cell lines, the Desmoplakin-mEGFP (DSP-GFP) and SOX2-GFP lines are particularly helpful for real-time culture monitoring of otic vesicle development (Roberts et al., 2017). To maintain a source of stem cells for differentiation, pluripotent stem cells were cultured on six-well plates with a coating of Vitronectin Recombinant Human Protein (Invitrogen, A14700) at a concentration of 0.5 µg/cm^2^. Cells were grown in Essential 8 Flex medium (Gibco, A2858501) supplemented with 100 µg/ml Normocin (hereafter, E8; InvivoGen, ant-nr-1). Culture media were replenished every other day. Around every 4 to 5 days of culture, cells reached 80% confluency and required passaging. Passaging procedures include removing adherent cell colonies using StemPro Accutase Cell Dissociation Reagent (hereafter, Accutase; Gibco, A1110501). Cells were passaged in a medium containing 10 µM Y27632 (hereafter, Y; Stemgent, 04-0012-02) to inhibit excessive apoptosis. Newly plated colonies received fresh E8 media within 24 h of passaging. Further information concerning the cell line used is available at https://www.allencell.org/cell-catalog.html. For the experimenter comparison experiments, we used the WA25 human ESC line (NIHhESC-12-0196) obtained from WiCell Research Institute.

### hPSC differentiation

Pluripotent hPSC colonies were detached similarly to the method used for passaging. Accutase was used for detachment and dissociation, and Y was used for apoptosis inhibition. An accurate measurement of cell concentration was obtained using an automated cell counter (Countess II Automated Cell Counter; Countess II, Life Technologies). Trypan Blue (Gibco, 15250061) was used at a ratio of 1:1 (Trypan Blue:cell suspension) to determine cell viability. Using the cell concentration measurement, the volume of cell suspension needed to obtain a concentration of 3500 cells per 100 µl of media was calculated, and 100 µl of this cell suspension was added per well in 96-well U-bottomed plates with a low-attachment surface (ThermoFisher Scientific, 174925). The plates were then centrifuged at 110 ***g*** for 6 min to form cell aggregates and incubated at 37°C with 5% CO_2_ for 48 h in E8 medium to maintain pluripotency. Therefore, our aggregation day was termed day −2. Halfway through the initial 48 h incubation period, 100 µl of fresh E8 medium was added to dilute Y and aid cell maintenance. After the 48 h incubation, all individual aggregates were collected and transferred to a new 96-well U-bottomed plate with 100 µl of differentiation media per well. This time point was termed day 0. The differentiation medium consisted of 100 µl of Essential 6 (supplemented with 100 µg/ml of Normocin; hereafter, E6; Gibco, A1516401) medium supplemented with 2% growth-factor reduced Matrigel (Corning, 354230), 10 µM SB431542 (Stemgent, 04-0010-05), 4 ng/ml basic-FGF (hereafter, FGF2; PeproTech, 100-18B) and 2.5 ng/ml BMP-4 (PeproTech, 120-05). After 3 days of incubation (day 3), 1 µM LDN-193189 (BMP inhibitor, Stemgent, 04-0074-02) and 250 ng/ml of FGF2 were added in a volume of 25 µl per well, thus increasing the volume in each well to 125 µl (final concentrations of 200 nM and 50 ng/ml of LDN and FGF2, respectively). After another 3 days of incubation (day 6), 75 µl of fresh E6 medium was added to each well, increasing the volume to 200 µl. On days 8 and 10 of culture, 100 µl of the culture medium from each well was removed and replaced with 100 µl of fresh E6 media containing 6 µM CHIR-99021 (CHIR; final concentration 3 µM in 200 µl; Stemgent, 04-0004-10). On day 12, cell aggregates were transferred to a floating culture system in 24-well low-attachment plates (ThermoFisher Scientific, 174930) in 500 µl organoid maturation medium (OMM) with 1% Matrigel. The plates were then incubated on an orbital shaker at a speed of 65 rpm to ensure that the aggregates did not adhere. The plates and orbital shaker were housed in an incubator maintained at 37°C with 5% CO_2_. OMM comprises Advanced DMEM/F12 (Gibco, 12634010) and Neurobasal (Gibco, 21103049) media at a ratio of 1:1, 1×GlutaMax (Gibco, 35050061), 0.5×B-27 Without Vitamin A (Gibco, 12587010), 0.5×N2 (Gibco, 17502048), 0.1 mM 2-mercaptoethanol (Gibco, 21985023) and 100 µg/ml Normocin. CHIR was added to OMM to maintain a consistent concentration of 3 µM. On day 15, half of the medium from each well was removed and replaced with fresh OMM containing 1% Matrigel and 3 µM CHIR. On and after day 18, the medium was replenished with fresh OMM every 3 days (day 18 to day 30) or every other day (after day 30). However, Matrigel was not added to the media beyond day 15. As the aggregates enlarged and increased in cell number, it was occasionally necessary to increase the media volume to 1 ml per well. Please see supplementary Materials and Methods for additional information concerning the differentiation protocol.

### Inner ear organoid dissociation for scRNA-seq

Cell aggregates were dissociated into single cells for single-cell transcriptomic analysis. Randomly selected cell aggregates were pooled at specified time points. Pooled cell aggregates were incubated in warm TrypLE (Gibco, 12605010) in an incubator at 37°C on a shaker at 65 rpm. During the TrypLE incubation, the cell aggregate mixture was triturated with a pipette using wide bore p1000 or p200 tips every 10 min, depending on aggregate size. By the end of the 10-60 min incubation, the tissues were dissociated into a single-cell suspension. Complete dissociation was confirmed by bright-field visualization at 10× using a Nikon TS2R. Cold 3% BSA solution (Sigma-Aldrich, A9576) in PBS was added to the single-cell suspension to halt TrypLE enzymatic activity. The suspension was then filtered through a 40 µm Flowmi cell strainer (Bel-Art, H13680-0040) to remove residual cell aggregated cells and debris. Three washes with ice-cold 3% BSA in PBS were performed to minimize ambient RNA in solution. The cells were then resuspended in ice-cold 3% BSA in PBS, and the filtering step was repeated. Trypan Blue staining and a Countess II Automated Cell Counter (Thermo-Fisher) were used to determine cell number and viability. Cell suspensions were diluted with ice-cold 3% BSA in PBS as necessary to obtain a final concentration of 1000 cells/µl. Cell viability was consistently 90% or higher.

### scRNA-seq cDNA library preparation and sequencing

Single-cell 3′ RNA-seq was performed using the Chromium platform (10x Genomics) and the NovaSeq 6000 sequencer (Illumina). Briefly, ∼17,000 cells (targeting 10,000 cells captured) per sample were added to a separately prepared single-cell master mix according to the Chromium Single Cell 3′ Reagent Kit v3 User Guide, CG000183 Rev C (10x Genomics, Inc). A Single Cell Chip B was loaded with the master mix, gel beads and partitioning oil as per the manufacturer's instructions. Once single-cell encapsulation was complete, cDNA was synthesized by reverse transcription, and Illumina libraries were prepared. At specific steps in the library preparation process, cDNA length and quality were analyzed with a Bioanalyzer. The final libraries were sequenced on an Illumina NextSeq 500 or NovaSeq 6000 using 28b plus 91b paired-end sequencing to a final read depth of >30,000 reads per cell.

### scRNA-seq data analysis

The 10x Genomics CellRanger 4.0.0 pipeline (http://support.10xgenomics.com/) was used to prepare data for analysis. Briefly, the CellRanger mkfastq command was used to demultiplex raw base sequenced calls into FASTQ files. The mkfastq command is a wrapper for bcl2fastq (http://support.illumina.com/) designed for multiplexed 10x libraries. The FASTQ files were then aligned to a reference GRCh38 genome using STAR aligner. Once the reads were aligned, they were traced to the individual encapsulated cell droplets of origin. Gene expression levels were analyzed based on the number of UMIs (unique molecular indices) detected in each cell. Filtered gene expression matrices were generated using CellRanger's count command. Cells with extreme (very low or very high, out of the 95% CI) library sizes, number of features and high content of mitochondrial reads (>10%) were excluded from further analyses. This quality control was performed over the merged dataset (Osorio and Cai, 2020). After removing these cells by filtering, gene expression levels were normalized for each cell by the total number of UMIs in the cell and multiplied by a scaling factor of 10,000. After log-transformation, Seurat3 was used for cell clustering and principal component analysis (PCA) to determine the most highly variable genes (Butler et al., 2018). Clusters of cells were visualized using Uniform Manifold Approximation and Projection (UMAP) plotting techniques. Specific gene markers to identify cell clusters were determined through differential expression analysis, comparing the expression of a specific cell cluster with the other cells in the dataset.

We made use of the Harmony package for R (Korsunsky et al., 2019) to integrate multiple datasets. Briefly, gene expression matrices were loaded as count matrices, and a pooled Seurat object was generated using the cbind command on the specified count matrices. Filtering, normalization and scaling were performed on this large Seurat object; however, the RunHarmony command was used to iteratively integrate cells to the point of convergence. For integrated datasets, harmony embeddings were used for all downstream analyses. Cell cluster identification was manually performed using cell type-specific marker genes. R packages ggplot2 and ggrepel (https://github.com/slowkow/ggrepel) were used to plot quantitative gene expression data. Violin plots (VlnPlot) and Feature plots (FeaturePlot) were used to analyze the specific gene expression patterns across clusters. Additional analyses were performed using scVelo (Bergen et al., 2020) and Symphony (Kang et al., 2021). A loom file was generated using the original velocyto implementation (La Manno et al., 2018), containing both the spliced and unspliced matrices. RNA velocity calculations were performed using all genes and the stochastic model, employing the Velociraptor wrapper of the scVelo Python library (Bergen et al., 2020). Vector fields were generated based on the baseline UMAP representation, with a resolution of 40 arrows per axis. Pseudotime values were also obtained from the velocity calculations. We used the Monocle3 v.0.0.9 package from Biocontainers (as a docker image) to conduct pseudotime analysis, helping us infer cell differentiation trajectories (Qiu et al., 2017; Trapnell et al., 2014). Initially, we transferred gene expression counts, cell and gene metadata from the Seurat object containing the atlas to a new Monocle3 cell_data_set object. To prepare the data, we computed the sequence of gene expression changes using 30 principal components and removed batch effects by using the sample id as an alignment group. Then, we generated a UMAP low-dimensional representation of the data using the reduce_dimensions function and fitted a graph describing the developmental process, ensuring all clustered partitions were joined. To create the pseudotime, we specified a root node at the iPSC populations and visualized it as a UMAP to observe the developmental progress through the projected trajectory. To ensure consistency, we transferred the computed coordinates produced by Seurat into the Monocle3 object, maintaining the same UMAP low-dimensional representation. Raw sequencing and output files from the 10x CellRanger pipeline have been deposited in GEO under accession number GSE188936.

### Immunohistochemistry

Samples were fixed in 4% paraformaldehyde (PFA, Electron Microscopy Sciences, 15710) and cryoprotected through a graded treatment process with 15% and 30% sucrose (Sigma-Aldrich, S0389). Samples were then embedded in cryomolds (Endwin Scientific) in tissue freezing medium (General Data Healthcare, TFM-C), snap-frozen at −80°C and sectioned at a thickness of 12 µm. Sections were blocked with 10% normal goat or horse serum, incubated with primary antibodies diluted in 3% normal goat or horse serum and then incubated with secondary antibodies in 3% normal goat or horse serum. Microscopy was performed with a Nikon A1R HD25 confocal microscope system. Three-dimensional reconstruction was performed with Imaris 8 (Bitplane) or the NIS Elements software package (Nikon). See Table S2 for a list of antibodies.

### Whole-mount clearing and imaging using SHIELD

Organoids imaged in Fig. 1E,F were rinsed once with PBS, then fixed with freshly prepared 4% PFA in 1×PBS at room temperature for 30 min on a shaker. Organoids were rinsed three times in PBS and transferred to ice-cold 12.5% SHIELD Epoxy (LifeCanvas Technologies, SH-Ex) in SHIELD Buffer (LifeCanvas Technologies, SH-BS) and incubated for 2 days on a gentle shaker at 4°C. Organoids were then transferred to pre-warmed SHIELD-ON solution (LifeCanvas Technologies, SH-ON) and incubated for 2 h at 37°C on a gentle shaker. Organoids were then washed extensively with fresh 1×PBS for 8 h (replaced every hour) and incubated with SHIELD Delipidation Buffer (LifeCanvas Technologies, DB) for 48 h at 55°C on a rotator. Immediately after delipidation, samples were washed extensively at room temperature with PBST (PBS, 0.1% Triton X-100 and 0.02% sodium azide) for 24 h. Samples were subsequently incubated with primary antibodies in 500 μl of 0.1% PBST buffer overnight on a shaker at room temperature. Samples were washed with 0.1% PBST three times over 3 h and incubated with secondary antibodies at room temperature for 4 h on a shaker, then washed three times with 0.1% PBST at room temperature over 3 h. Before imaging, samples were equilibrated with a 1:1 mix of Easy-Index Matching Solution (LifeCanvas Technologies, EI-Z1001) and 1×PBS for 4 h at 37°C. Solution was then replaced with a pure immersion medium (100% Easy-Index Matching Solution) for at least 6 h at 37°C. Images were acquired using a Nikon A1R HD25 confocal microscope system.

### RNAScope

Samples were initially processed and sectioned as described for the immunohistochemical analysis. The samples were then further processed according to the RNAScope Multiplex Fluorescent Reagent Kit v2 Assay User Manual. Briefly, samples were incubated at 60°C for 30 min, postfixed in 4% paraformaldehyde for 15 min, dehydrated in increasing ethanol concentrations and incubated in hydrogen peroxide. Samples were then incubated with target probes for 2 h at 40°C, and subsequently with amplification and fluorophore reagents. After DAPI application and coverslip-mounting, samples were imaged with a Nikon A1R HD25 confocal microscope system.

## Supplementary Material

10.1242/develop.201871_sup1 Supplementary information
