# Supplementary material for: Mapping oto-pharyngeal development in a human inner ear organoid model
Source: Development. 2023 Oct 5;150(19):dev201871. doi: 10.1242/dev.201871 (PMC10698753; doi:10.1242/dev.201871)
Supplement: Supplementary information [file develop-150-201871-s1.pdf]

Supplementary Materials and Methods

TECHNICAL NOTE

Extended discussion of methodology development and troubleshooting

Since our last publication on the inner ear organoid protocol (Koehler et al., 2017), we have made several modifications and validated the protocol with various cell lines (van der Valk et al., 2023). However, this report focuses specifically on the use of the WTC cell line. The detailed methods can be found in (Zhang et al., 2021).

Basal medium optimization: We updated the composition of certain media elements used during differentiation. Previously, we used a Chemically Defined Medium (CDM). However, we found that batch-to-batch variability caused by pipetting errors or reagent stability made CDM unsuitable. Therefore, we substituted individually prepared CDM for commercially available Essential 6 (E6) media (Gibco, A1516401) due to its fully defined and stable components. With E6 media as a basal media, we can supplement with additional small molecule and growth factor components tailored to our specific needs.

Matrigel optimization: We optimized our use of Matrigel (Corning Matrigel Growth Factor Reduced, 354230) as a culture component. Previously, we embedded cell aggregates in droplets of 100% Matrigel on the bottom of a petri dish and then submerged the droplets in medium. However, this can be a time-consuming process and leads to significant Matrigel use. We have found that we can introduce Matrigel to cell aggregates by adding Matrigel to the media at a 1% concentration and then suspending cell aggregates in floating culture. This approach has shown robust inner ear organoid generation.

Long-term culture format: We previously used a spinner flask for long-term culture of cell aggregates but have switched to using individual wells of a low adhesion 24-well plate (Thermo Scientific, 174930) for long-term culture. This modification allows us to follow individual aggregates over time and makes it easier to track experimental groups. We place a single aggregate in each well and then place the plate on an orbital shaker.

CHIR 99021 utilization: We updated the timing and concentrations of our CHIR-99021 (CHIR; Stemgent, 04-0004-10) treatments in culture. On day 8, we treat cell aggregates with 3 μM of CHIR. Then, on day 10, we add CHIR again at the same concentration. On day 12, we transition to floating culture but ensure that the 3 μM concentration of CHIR is maintained as aggregates are transitioned to fresh media. On day 15, we perform a half-medium change, but also add CHIR to maintain the concentration at 3 μM. Finally, on day 18 of culture, we perform a half-medium change but do not supplement the media with CHIR. We do not use CHIR as a media component again during continued long-term culture beyond day 18.

Development • Supplementary information

QUALITY CONTROL

Received datasets:

We received pre-processed matrices generated from CellRanger for the ten timepoints (day 0, 3, 6, 8, 10C, 13C, 18, 24, 30, 36). Before quality control, we found problematic patterns (bimodal number of genes and high mitochondrial content) in sample of day 0. All other samples look normal.

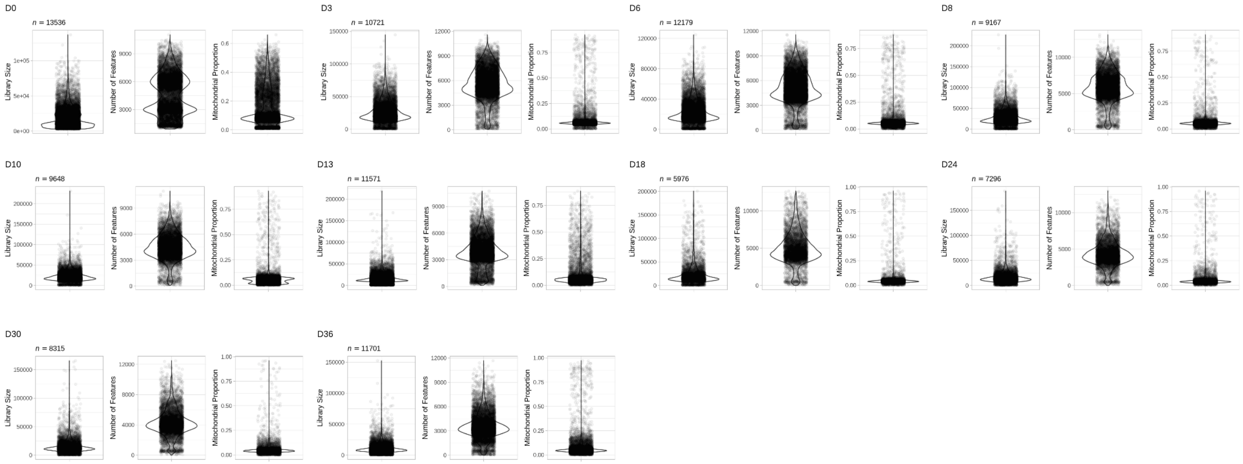

Data Merged

We filtered out cells with extreme (very low or very high, out of the 95% CI) library sizes, number of features and high content of mitochondrial reads (> 10%). Quality control was performed over the merged dataset. Plots show the distribution of the merged data before (left) and after (right) quality control.

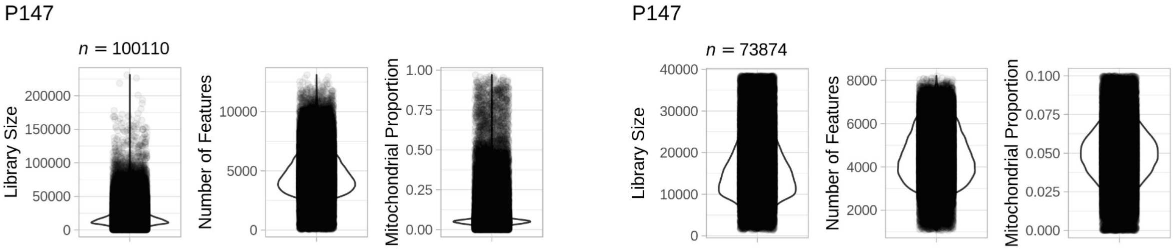

3136 identified doublets were removed

Development • Supplementary information

Data Integration

The number of dimensions (n = 10) from the principal component analysis (PCA) used to generate the low dimensional representation of the data was selected using the elbow plot displayed on the left side of the slide. On the middle of the slide, we display the generated low dimensional representation of the data before integration (batch effect removal), while on the right side we show the data after integration using Harmony.

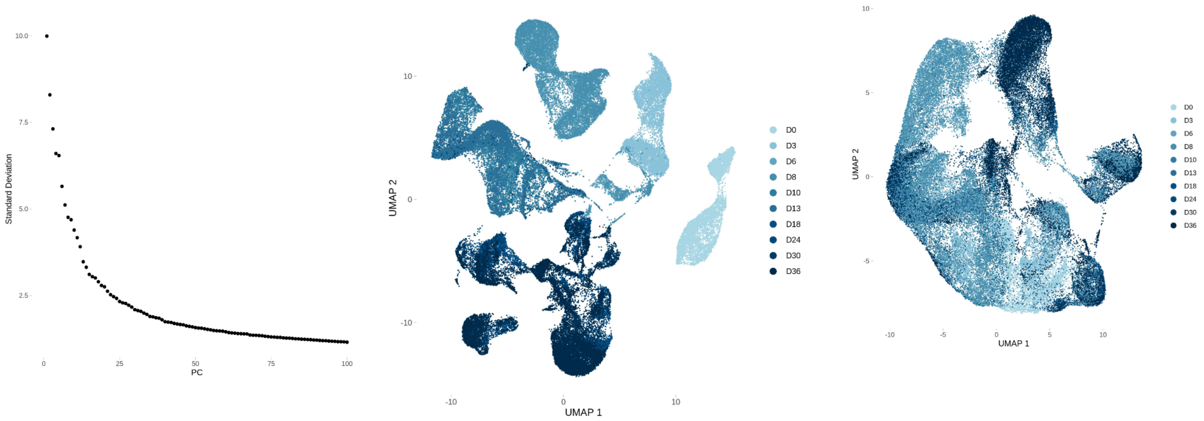

Testing 10, 30, 50 and 100 principal components

We tested selecting 10, 30, 50 and 100 principal components to perform the Harmony integration and UMAP low dimensional representation. We found that adding more components generates cluster of cells from individual time points, representing differentiation of the inner ear organoids over time.

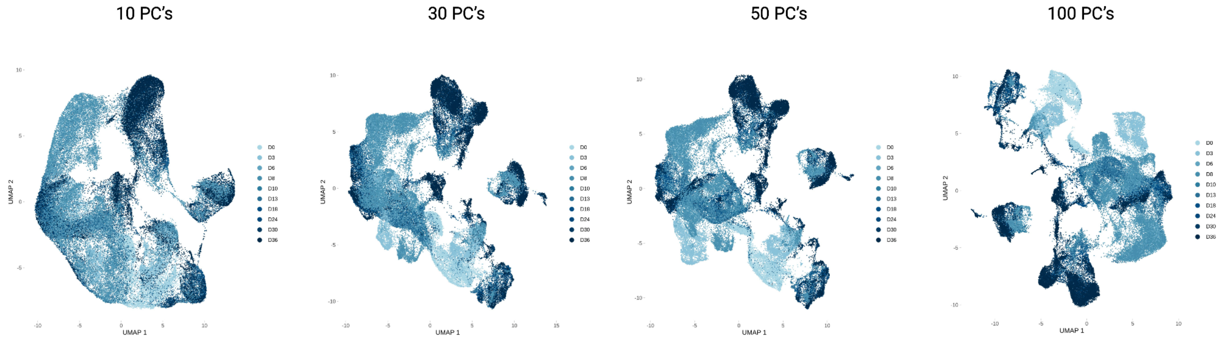

Development • Supplementary information

Spectral clustering - 10PCs

We performed spectral clustering using the top 10 harmony dimensions and a resolution of 0.5. We identified 18 subpopulations of cells (left) for which we identified the top 5 marker genes and displayed them on a dot plot (right).

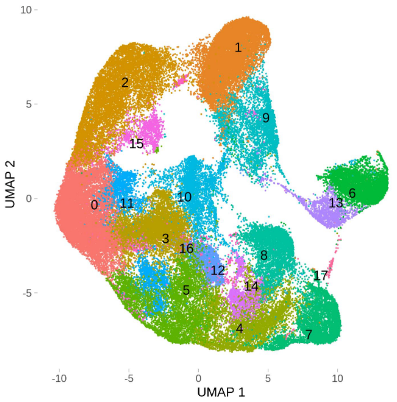

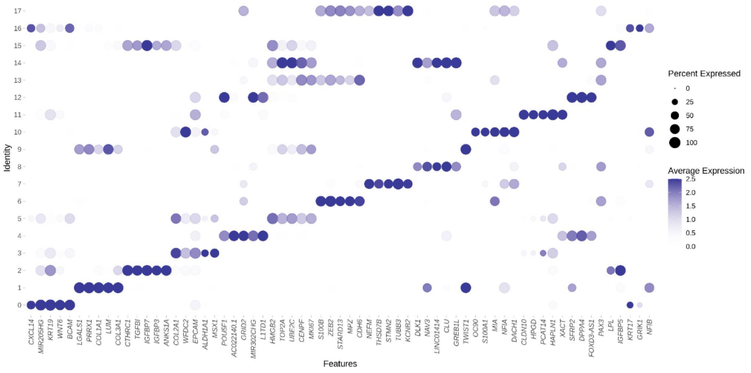

Marker genes - 10 PCs

We received a set of marker genes to perform the annotation of the data. On the right side of the slide is a dot plot displaying the clusters expressing the gene and on the right UMAP plots showing the spatial distribution of the marker in the sample

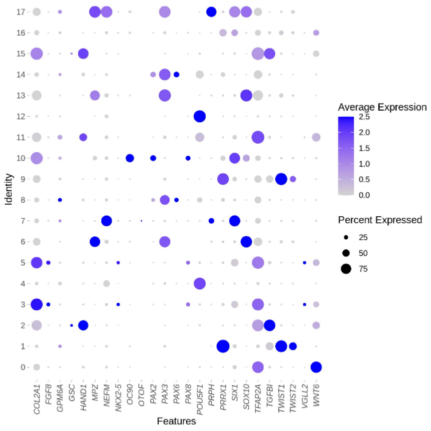

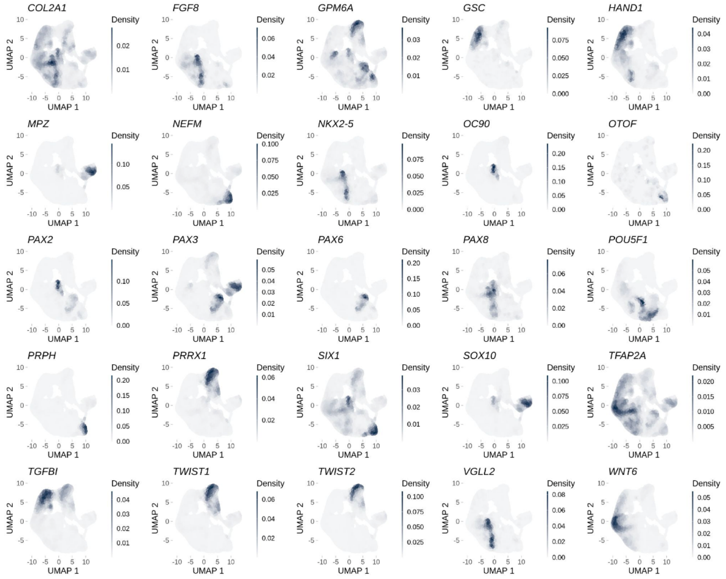

Development • Supplementary information

Spectral clustering - 30PCs

We performed spectral clustering using the top 30 harmony dimensions and a resolution of 0.5. We identified 23 subpopulations of cells (left) for which we identified the top 5 marker genes and displayed them on a dot plot (right).

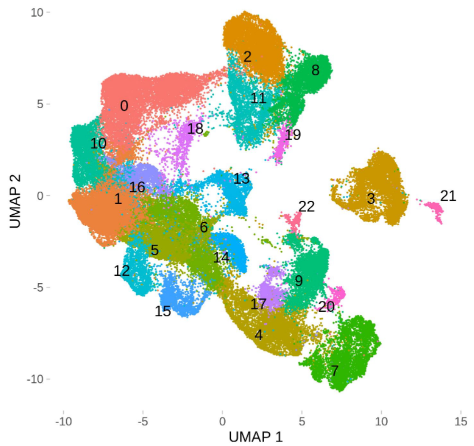

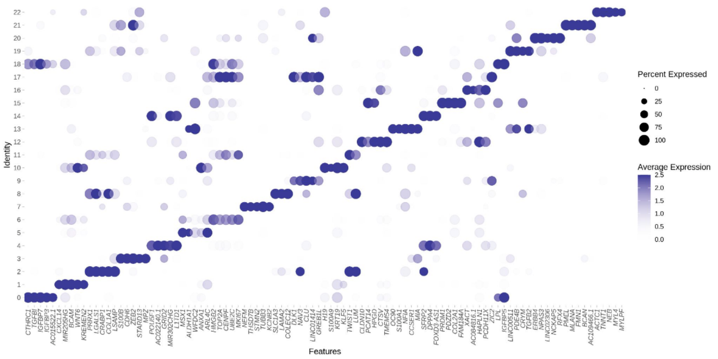

Marker genes - 30 PCs

We received a set of marker genes to perform the annotation of the data. On the right side of the slide is a dot plot displaying the clusters expressing the gene and on the right UMAP plots showing the spatial distribution of the marker in the sample

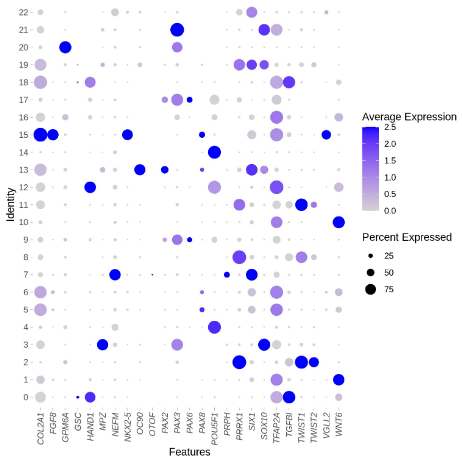

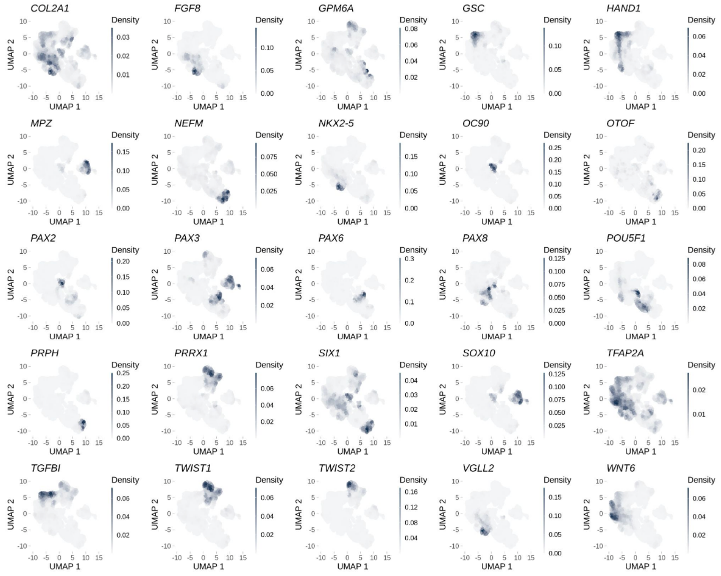

Development • Supplementary information

Spectral clustering - 50PCs

We performed spectral clustering using the top 50 harmony dimensions and a resolution of 0.5. We identified 20 subpopulations of cells (left) for which we identified the top 5 marker genes and displayed them on a dot plot (right).

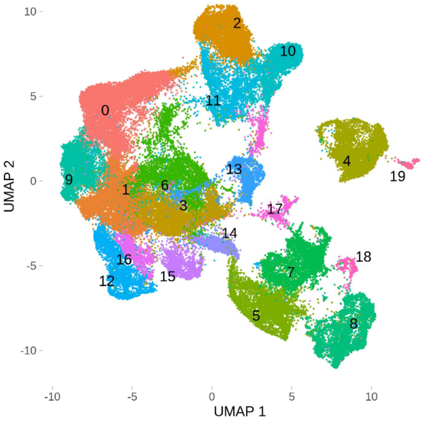

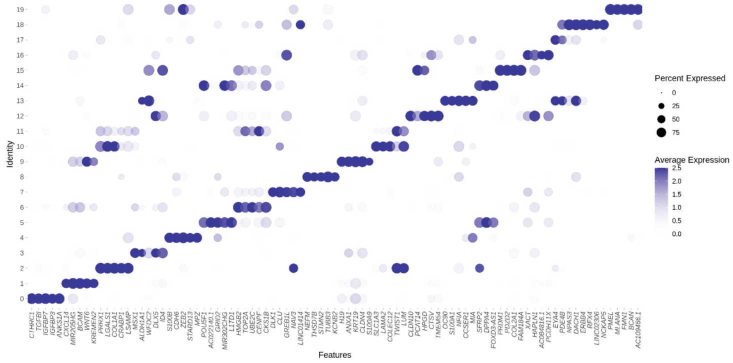

Marker genes - 50 PCs

We received a set of marker genes to perform the annotation of the data. On the right side of the slide is a dot plot displaying the clusters expressing the gene and on the right UMAP plots showing the spatial distribution of the marker in the sample

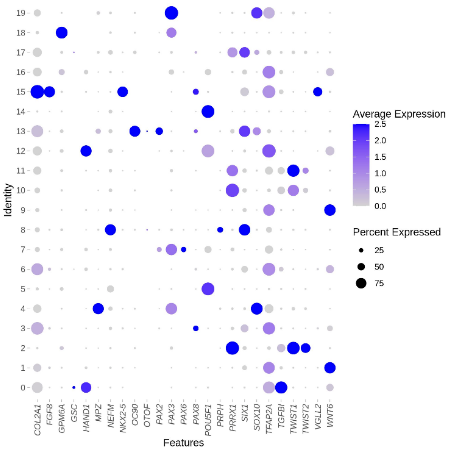

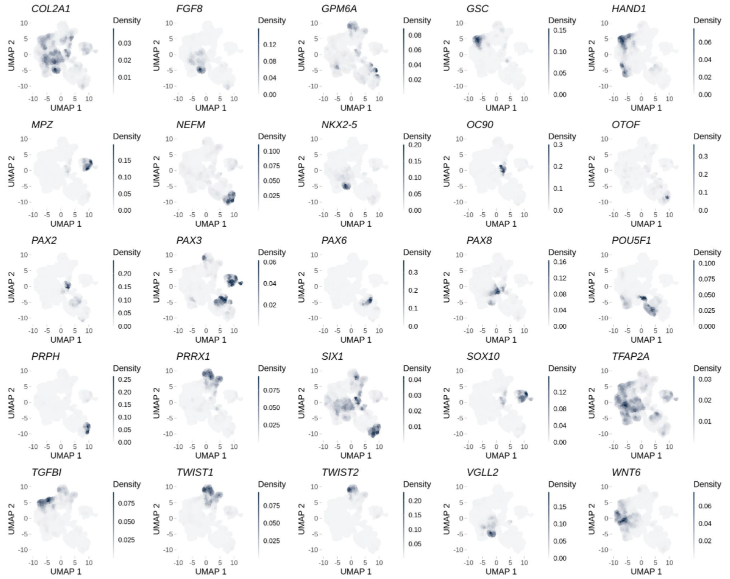

Development • Supplementary information

Spectral clustering - 100PCs

We performed spectral clustering using the top 100 harmony dimensions and a resolution of 0.5. We identified 23 subpopulations of cells (left) for which we identified the top 5 marker genes and displayed them on a dot plot (right).

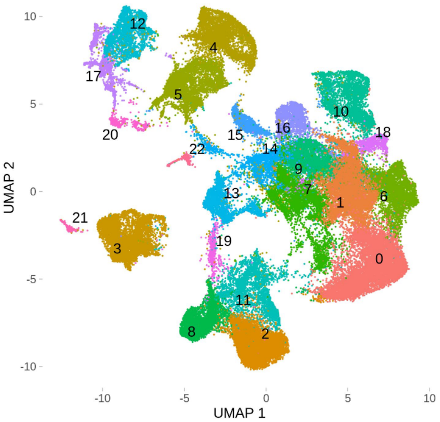

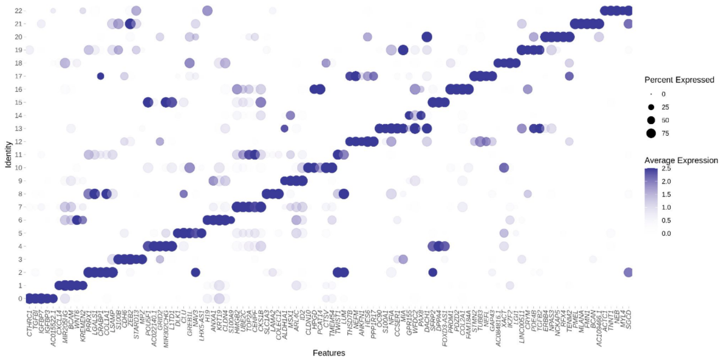

Marker genes - 100 PCs

We received a set of marker genes to perform the annotation of the data. On the right side of the slide is a dot plot displaying the clusters expressing the gene and on the right UMAP plots showing the spatial distribution of the marker in the sample

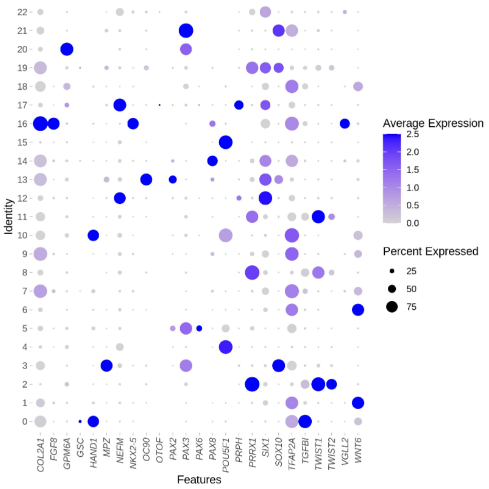

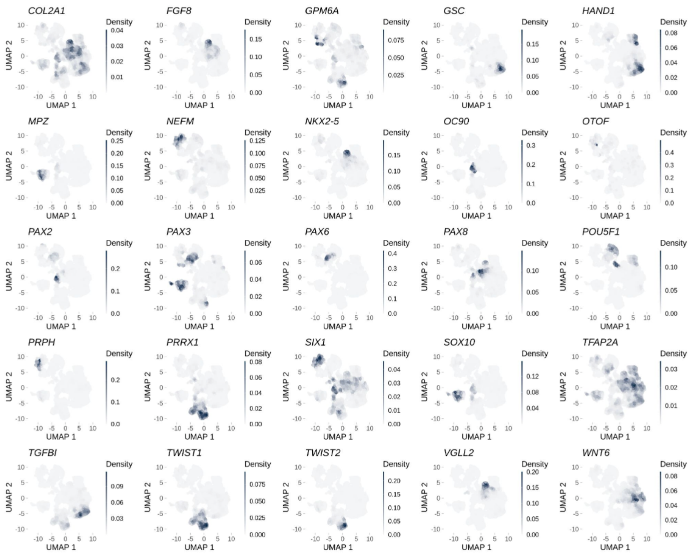

Development • Supplementary information

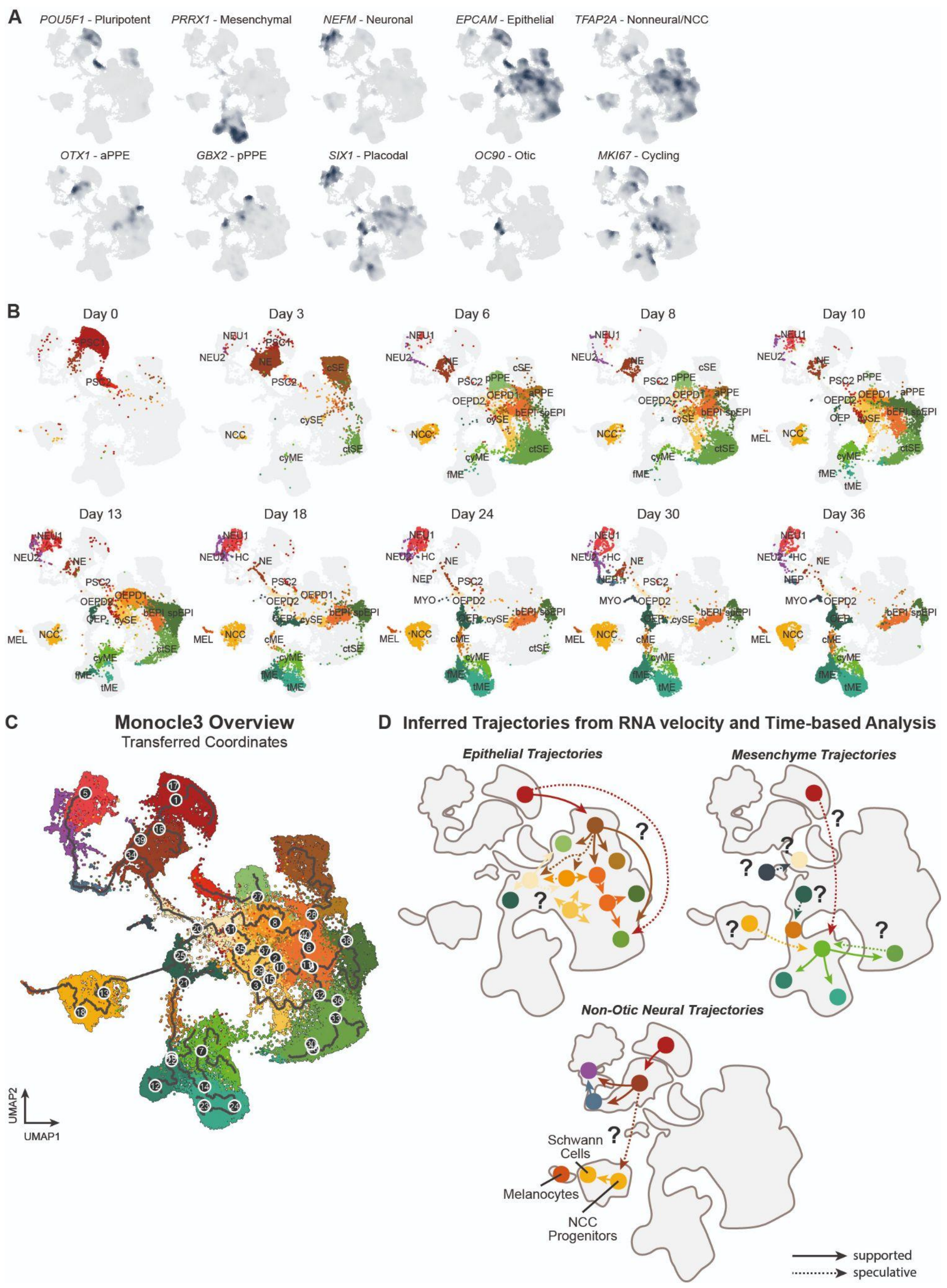
A
POU5F1 - Pluripotent
PRRX1 - Mesenchymal
NEFM - Neuronal
EPCAM - Epithelial
TFAP2A - Nonneural/NCC
OTX1 - aPPE
GBX2 - pPPE
SIX1 - Placodal
OC90 - Otic
MKI67 - Cycling
B
Day 0
Day 3
Day 6
Day 8
Day 10
Day 13
Day 18
Day 24
Day 30
Day 36
C
Monocle3 Overview
Transferred Coordinates
UMAP2
UMAP1
D
Inferred Trajectories from RNA velocity and Time-based Analysis
Epithelial Trajectories
Mesenchyme Trajectories
Non-Otic Neural Trajectories
Schwann Cells
Melanocytes
NCC Progenitors
supported
speculative

Fig. S1. Key Gene Expression, Trajectories Revealed by Time Point and Pseudotime Analyses of the Inner Ear Organoid Developmental Atlas (IODA). (A) Marker gene expression within the day 0 to day 36 integrated dataset, depicting expression of pluripotent, mesenchymal, neuronal, epithelial, nonneural, placodal, otic, and cycling markers. (B) UMAP plotting the data per experimental time point. (C) Monocle3 analysis results showing the cellular transitions in the UMAP. (D) Trajectories revealed by analysis of scVelo and Monocle3 pseudotime and experimental time points (see also Fig. S2). Solid arrows indicate trajectories supported by RNA velocity vectors. Dotted arrows represent speculative trajectories based on time-based clustering and analysis of cluster positioning at different PC levels. "?" indicates transitions of lingering ambiguity that should be examined using higher resolution timepoint sampling or spatial transcriptomics. aPPE: anterior pre-placodal ectoderm; bEPI: basal epidermis; ctSE: surface ectoderm; cySE: cycling surface ectoderm; tME: mesenchyme; fME: mesenchyme; HC: hair cells; ME: mesenchyme; MEL: melanocytes; MYO: myocytes; NCC: neural crest; NE: neuroectoderm; NEP: neuroepithelial cells; NEU: neurons; OP: otic epithelium; OEPD: otic-epibranchial progenitor domain; PSC: pluripotent stem cell; sCE: surface ectoderm; spEPI: suprabasal and peridermal epidermis; pPPE: posterior pre-placodal ectoderm

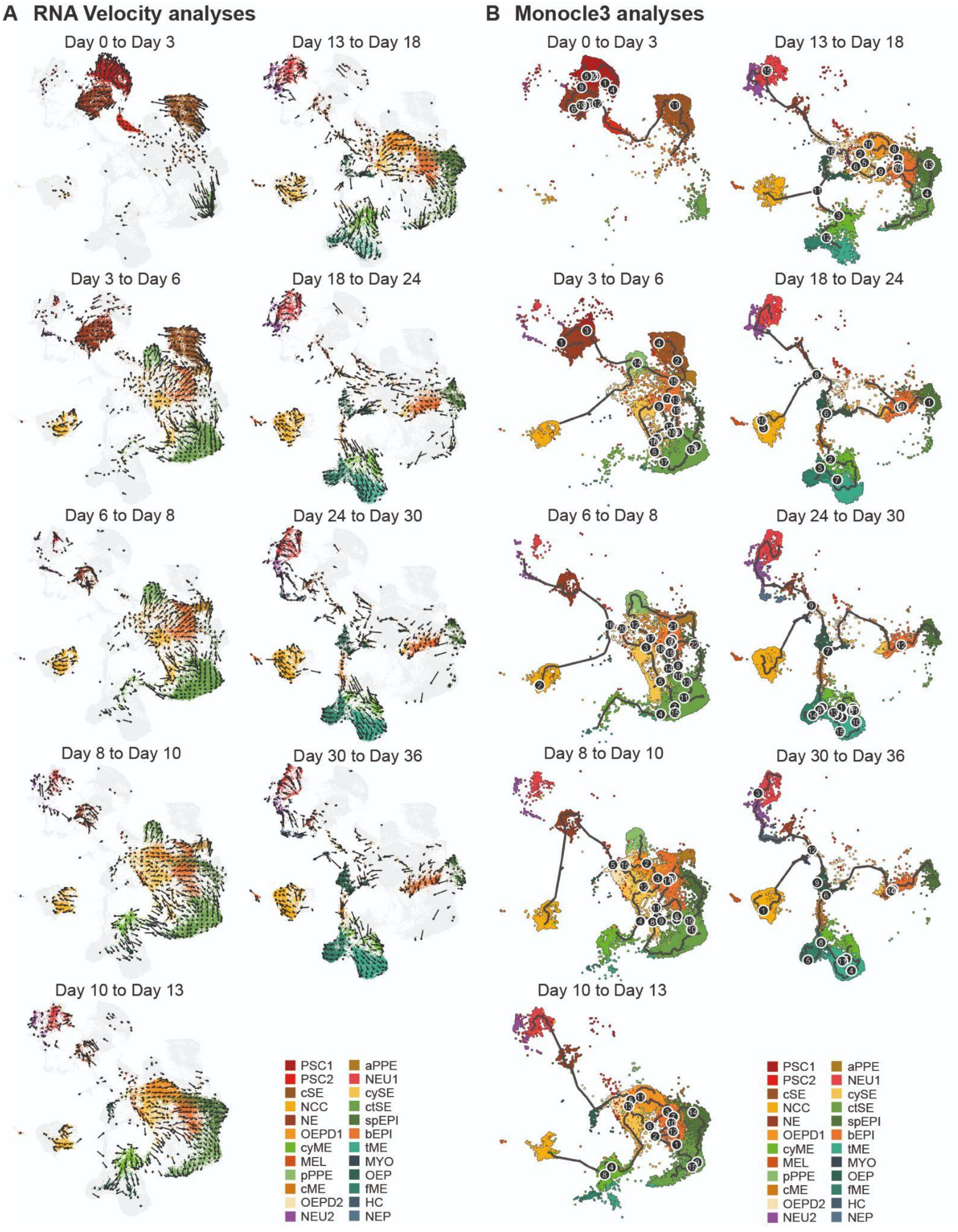

Fig. S2. IODA Pseudotime Analysis on Limited Time Intervals. (A) scVelo analyses combining two consecutive experimental time points. (B) Monocle3 analyses combining two consecutive experimental time points. aPPE: anterior pre-placodal ectoderm; bEPI: basal

Development • Supplementary information

epidermis; ctSE: surface ectoderm; cySE: cycling surface ectoderm; tME: mesenchyme; fME: mesenchyme; HC: hair cells; ME: mesenchyme; MEL: melanocytes; MYO: myocytes; NCC: neural crest; NE: neuroectoderm; NEP: neuroepithelial cells; NEU: neurons; OP: otic epithelium; OEPD: otic-epibranchial progenitor domain; PSC: pluripotent stem cell; sCE: surface ectoderm; spEPI: suprabasal and peridermal epidermis; pPPE: posterior pre-placodal ectoderm

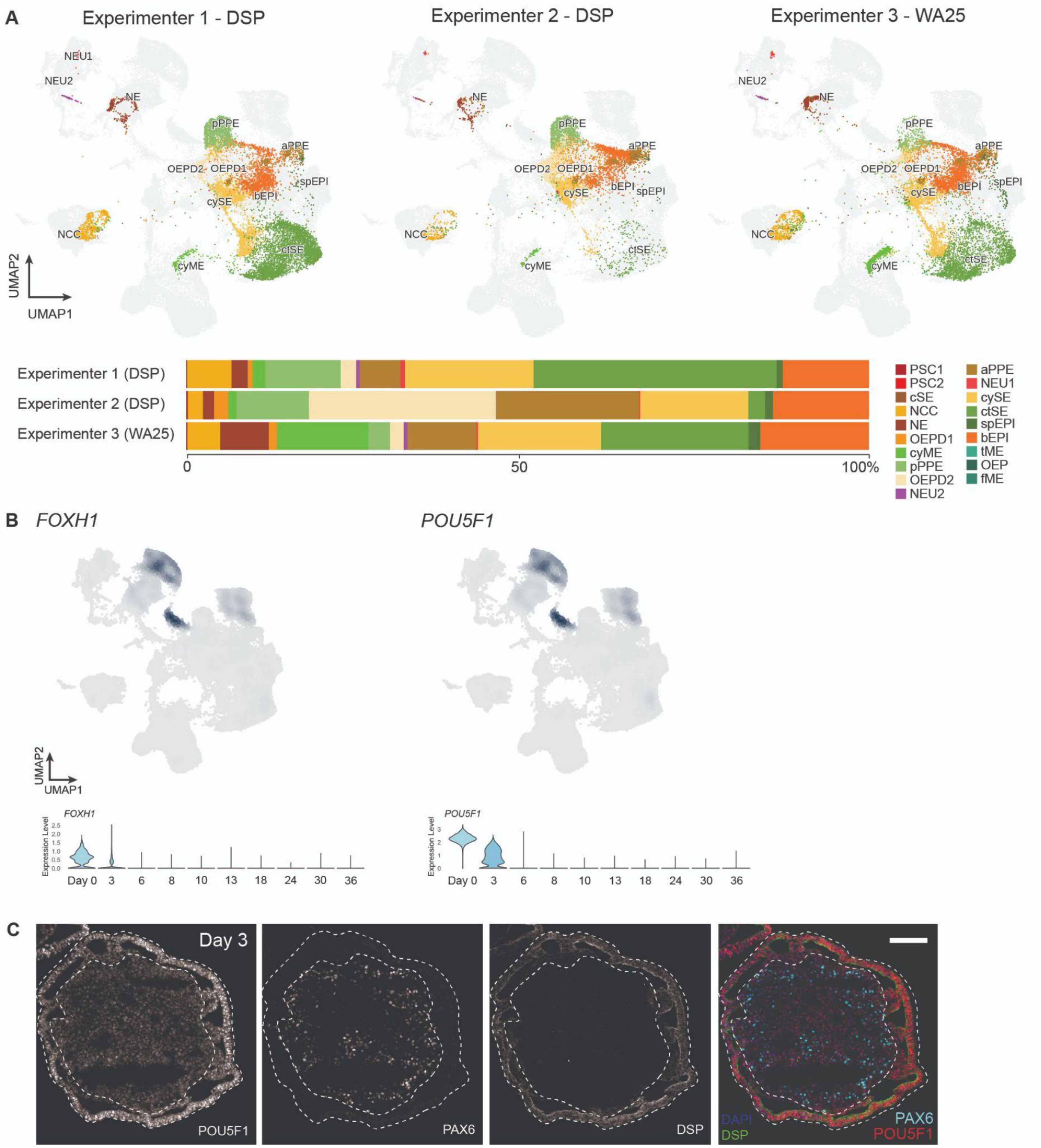

Fig. S3. IODA Reproducibility and Pluripotency Markers. (A) UMAPs as result of mapping by Symphony of day 6 organoids from two experiments using the hiPSC line WTC-DSP and one experiment using the WA25 hESC line. The bar plot displays the relative cell type contribution per experiment. By comparing cell type contributions across multiple experimenters and cell lines, it was clear that there were differences

Development • Supplementary information

between the proportions of cell types between experimenters and cell lines, but the same broad cell types were represented across experimenters and cell lines. (B) Density plots displaying expression of pluripotency markers *POU5F1* and *FOXH1* (day 0 through day 36 data) with corresponding violin plots displaying decreased expression over the time points. (C) POU5F1 expression in the outer surface ectoderm layer (DSP^{+}) in day 3 organoids. Scale bar 100 μm.

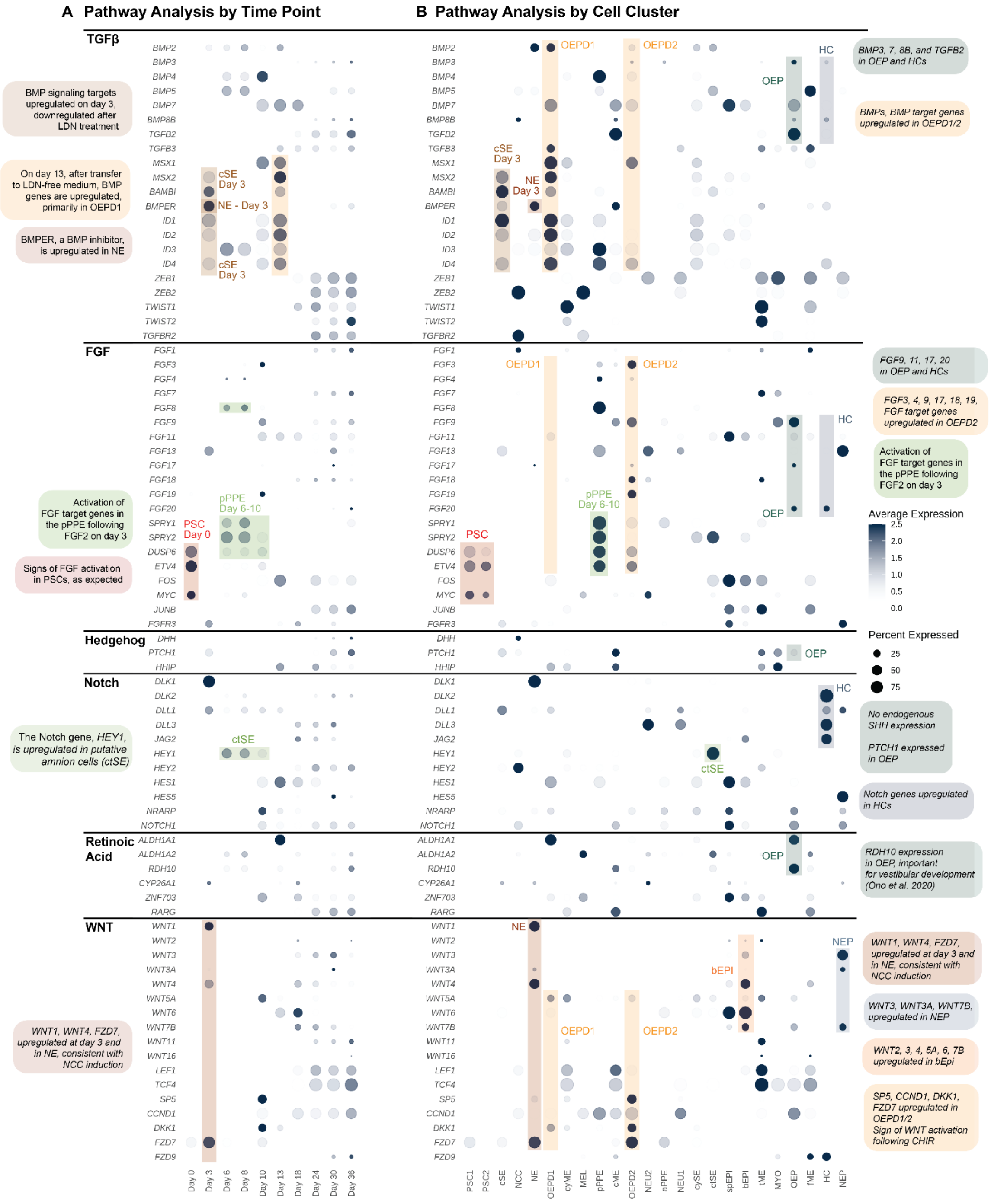

Fig. S4. Cell Signaling Analyses Across Time Points and Cell Types. (A) Gene expression of targets of the TGFβ, FGF, Hedgehog, Notch, retinoic acid, and WNT signaling pathways in the whole dataset per time point. (B) Gene expression per cell type of members of the TGFβ, FGF, retinoic acid, Hedgehog, Notch, and WNT signaling pathways per cell cluster. Signaling pathway members that showed no expression were excluded. Annotations highlight a select group of expected and novel insights. *RDH10* expression citation (Ono et al., 2020).

Development • Supplementary information

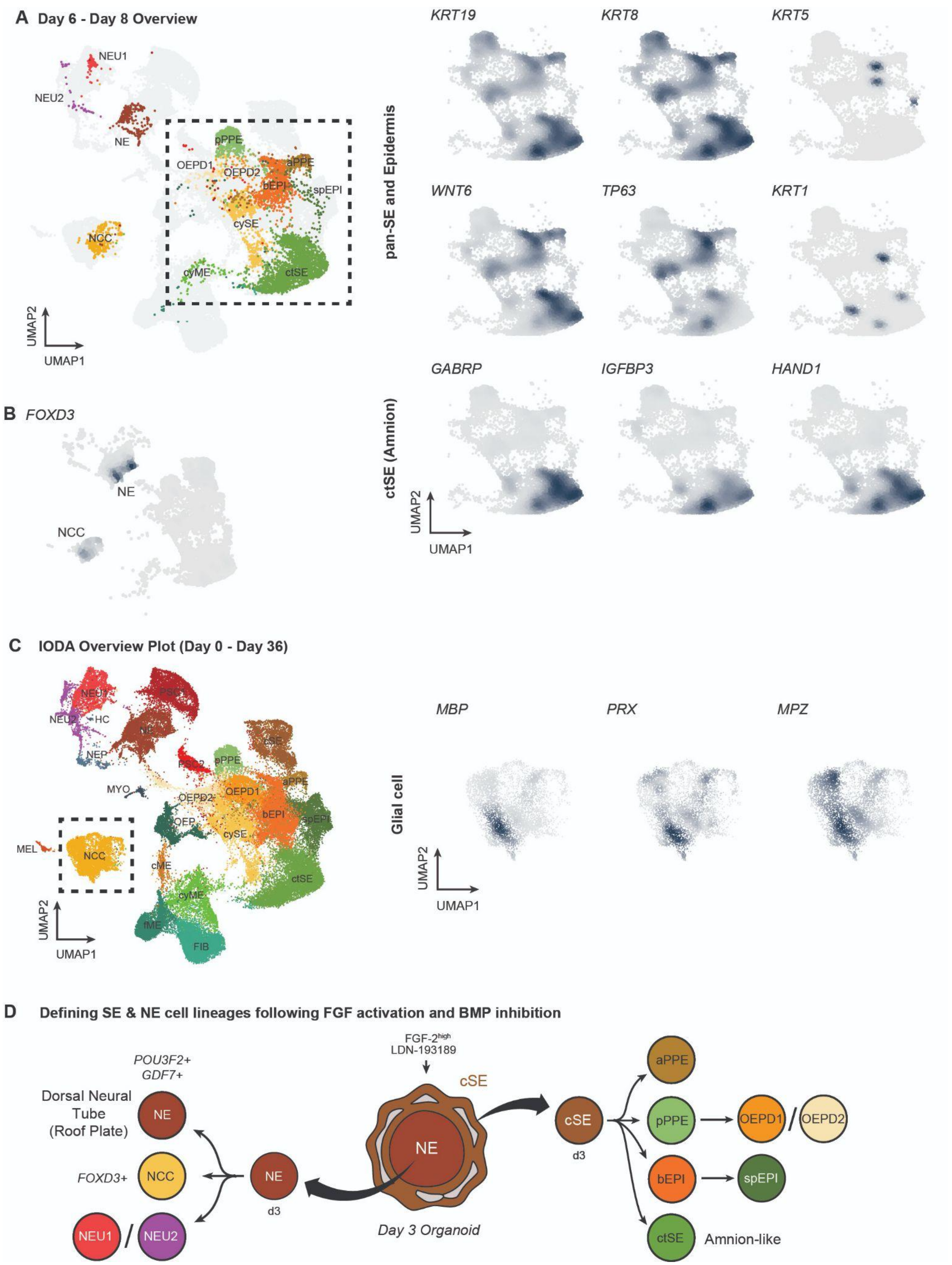
A Day 6 - Day 8 Overview
NEU1
NEU2
NE
pPPE
OEPD1
OEPD2
aPPE
bEPI
spEPI
cySE
NCC
cyME
ctSE
UMAP2
UMAP1
KRT19
KRT8
KRT5
pan-SE and Epidermis
WNT6
TP63
KRT1
GABRP
IGFBP3
HAND1
ctSE (Amnion)
B FOXD3
NE
NCC
C IODA Overview Plot (Day 0 - Day 36)
MBP
PRX
MPZ
Glial cell
NEU1
NEU2
HC
NEP
PSC1
PSC2
cSE
MYO
OEPD2
OEPD1
OEP
MEL
NCC
cME
cyME
fME
FIB
ctSE
D Defining SE & NE cell lineages following FGF activation and BMP inhibition
POU3F2+
GDF7+
Dorsal Neural Tube (Roof Plate)
FOXD3+
NEU1
NEU2
d3
FGF-2high
LDN-193189
Day 3 Organoid
Amnion-like

Fig. S5. Epidermal and Amnion Markers at Days 6 and 8, NE to NCC Transition, Glial Marker Expression in NCC, and the Working Model of Lineage Segregation. (A) Density plots displaying expression of marker genes for surface ectoderm and epidermis, as well as marker genes for amnion cells in day 6 to day 8 data. (B) Shared FOXD3 expression between NE and NCC in day 3 through day 6 data. (C) Density plots displaying expression of marker genes for glial cells in the NCC cluster (day 0 to day 36). (D) Illustration demonstrating the development of surface ectoderm and neuroectoderm derivatives after supplementing day 3 organoids with FGF and inhibiting BMP.

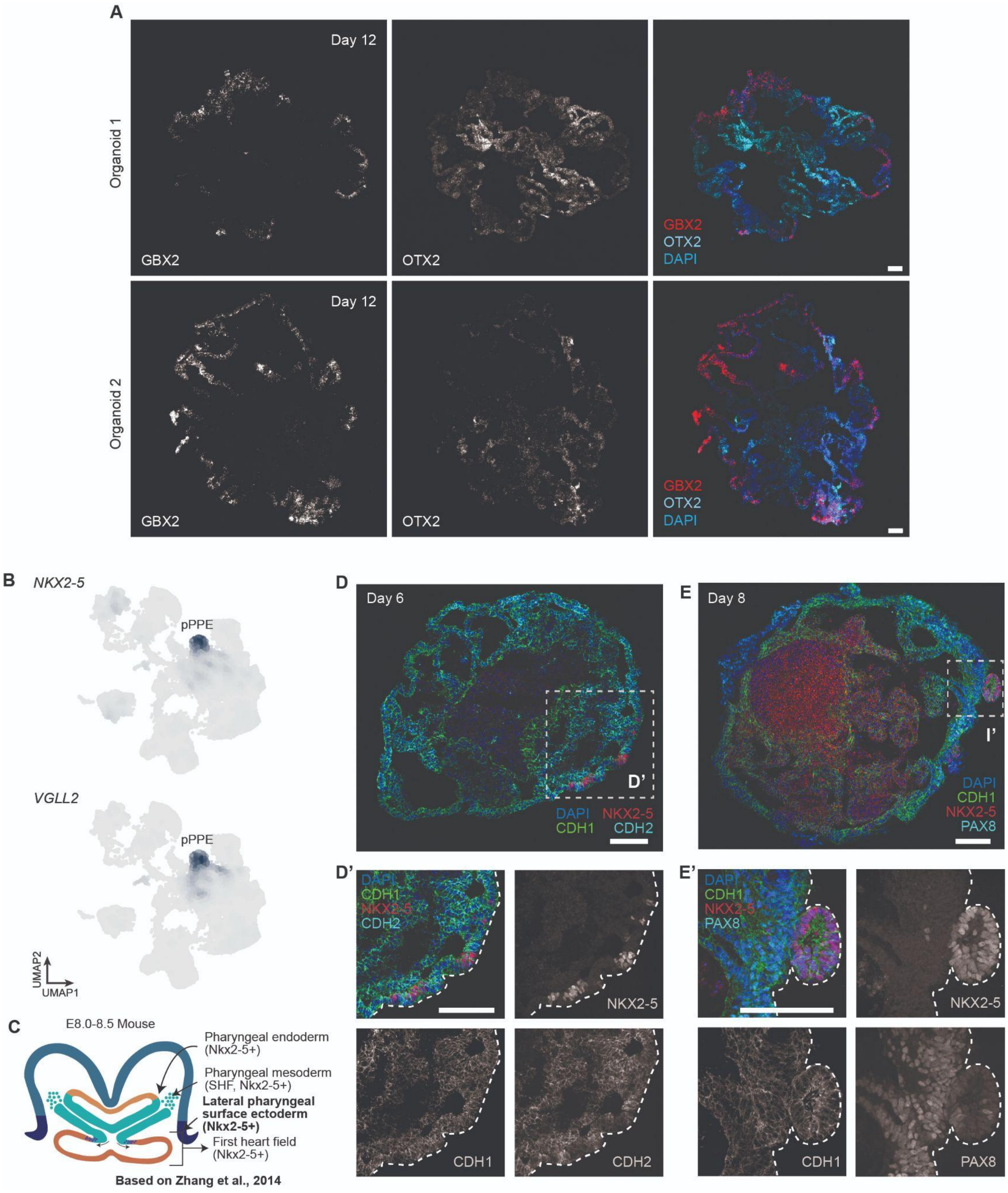

Fig. S6. Spatial Analysis of Placodal and Pharyngeal Marker Genes and Proteins. (A) Distinct expression of posterior placodal marker *GBX2* and anterior placodal marker *OTX2* in day 12 organoids by RNAScope. (B) Density plots showing expression of pharyngeal markers *NKX2-5* and *VGLL2* in the pPPE cluster (day 0 to day 36 data). (C) Illustration of NKX2-5 pharyngeal expression in the developing mouse embryo, based on (Zhang et al., 2014). (D)

Development • Supplementary information

Protein expression of NKX2-5 in day 6 organoids, displaying pharyngeal-like cells in CDH1^{+}/CDH2^{+} outer layer (D') composed of developing surface ectoderm to pre-placodal cells. (E) IHC on day 8 organoids reveal a similar expression with NKX2-5 expression in PAX8^{+} pre-placodal outer layer. Scale bars 100 µm.

Development • Supplementary information

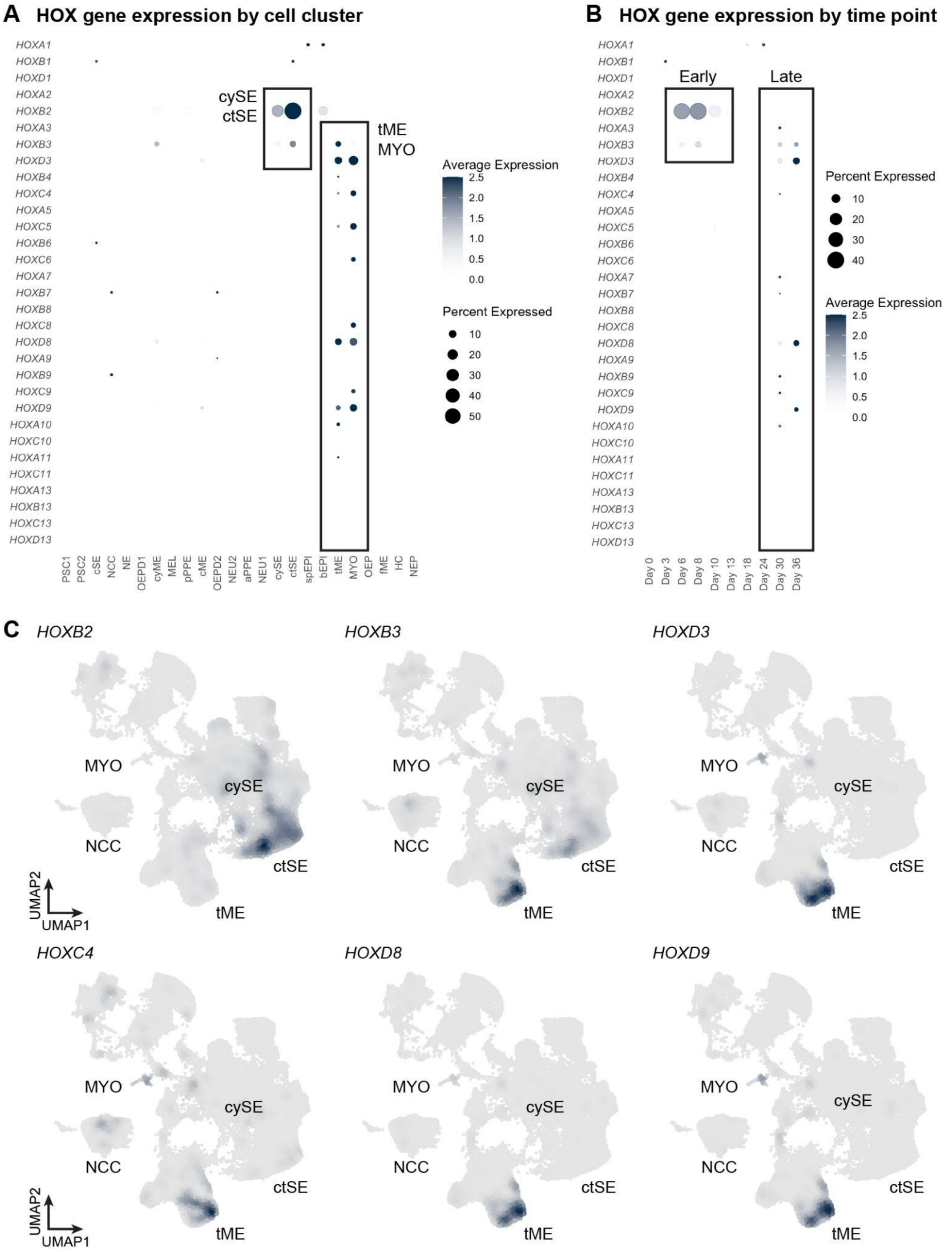

Development • Supplementary information

Fig. S7. HOX Gene Analysis. (A) HOX gene expression analysis showed that the mesenchyme (cySE, ctSE, tME, and MYO) clusters expressed low levels of HOX genes, which can help to determine the axial level of development. Additionally, the lack of HOX gene expression in the NCC was consistent with past analyses of the lack of HOX gene expression in cranial neural crest cells. (B) The HOX gene expression in the cySE and ctSE clusters started around day 6 and decreased around day 8, whereas HOX expression in tME and MYO clusters increased around day 30-36 of the culture. (C) Density plots of HOX gene expression in the day 0 through day 36 data.

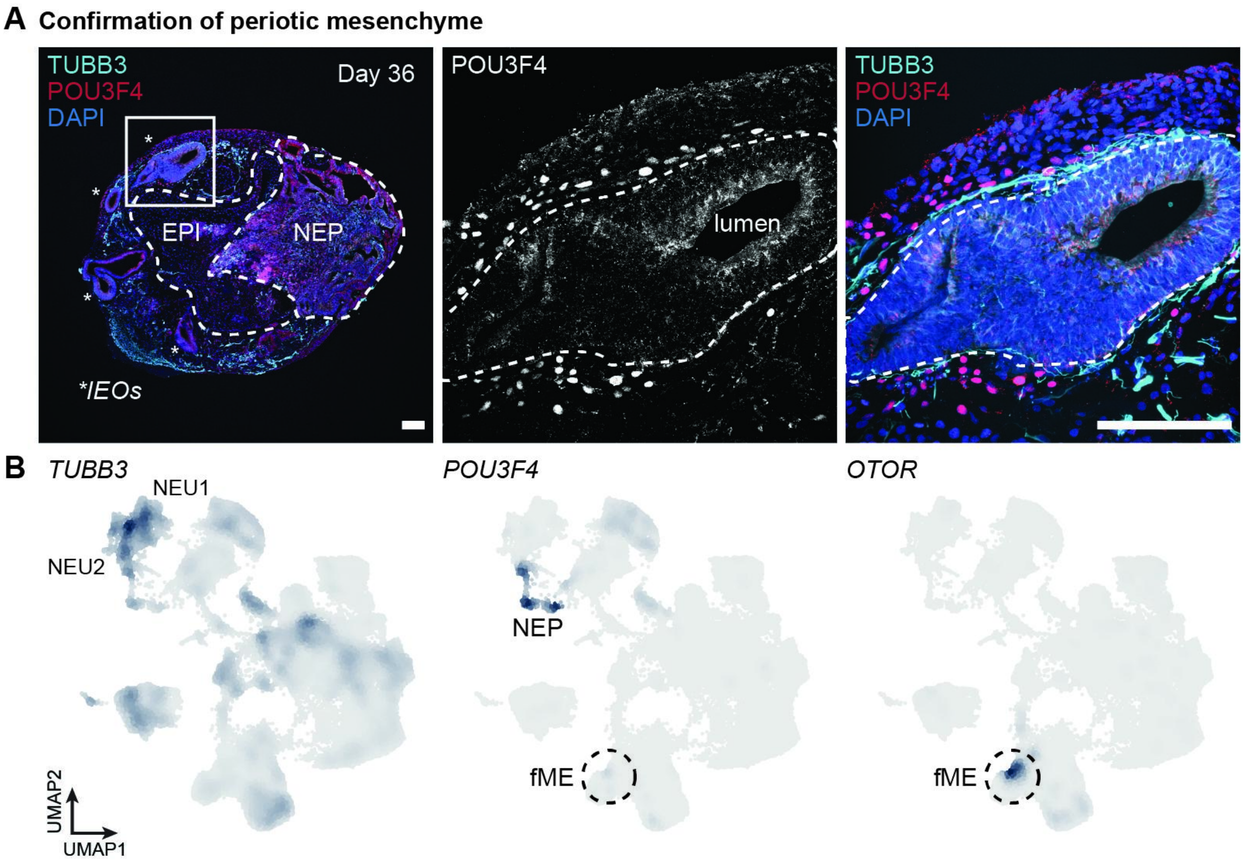

Fig. S8. Spatial Analysis of Periotic Mesenchyme. (A) Considering that POU3F4^{+} mesenchyme plays an important role in axon guidance during inner ear development, we stained organoid samples at day 36 for the neuronal marker, TUBB3, and POU3F4. A higher magnification image revealed TUBB3^{+} axons and POU3F4^{+} mesenchyme surrounding a developing inner ear organoid. (B) In addition to immunohistochemistry analysis, we also examined transcript expression and found *OTOR* and *POU3F4* expression in the fME mesenchymal cluster. *TUBB3* was expressed in the neuronal cell cluster. Note the POU3F4 expression in NEP and the CNS-like portion of the NEU cluster, consistent with its role in neurodevelopment. Scale bars 100 µm.

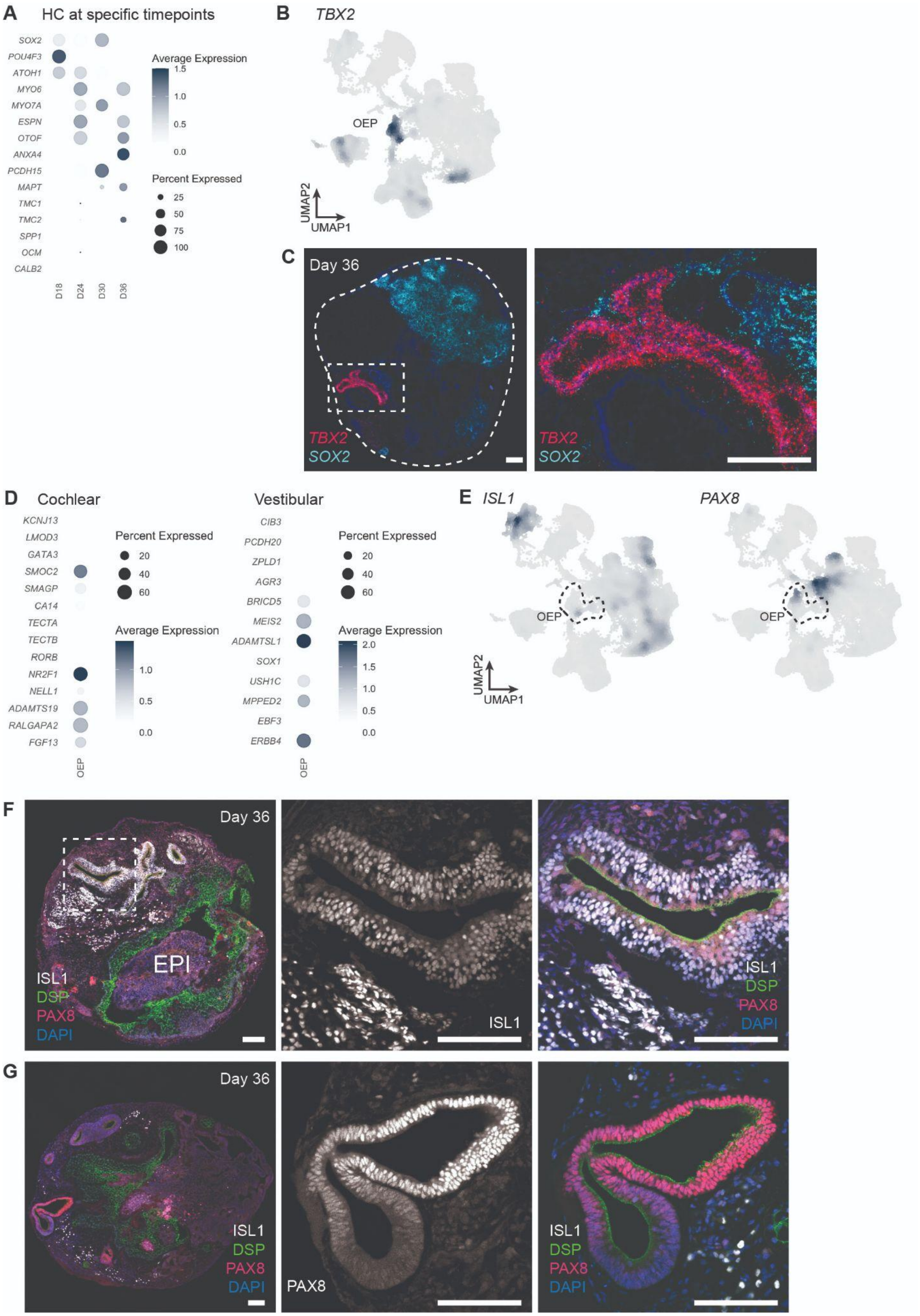

Development • Supplementary information

Fig. S9. HC Maturation and Segregation of OEP in Cochlear versus Vestibular and Sensory versus Nonsensory Domains. (A) Dynamic expression of genes in hair cells that are known to have a temporal expression during hair cell development. (B) Considering the role that TBX2 plays in inner ear patterning, we analyzed our dataset for *TBX2* expression and found otic epithelium expression. To confirm otic *TBX2* expression, we performed in situ hybridization with the RNAScope platform on day 36 samples (C) and found *TBX2* expression throughout the epithelium. (D) Cochlear versus vestibular marker gene expression in the OEP. (E) Within the OEP cell cluster of day 0 through day 36, there appears to be a spectrum of expression: the lower area expresses *PAX8,* and the upper portion *ISL1*. This highlighted the diversity of cell types contained in the otic epithelium cell cluster and suggested a broad spectrum of otic vesicle patterning was represented. To confirm the transcriptomic analysis, we performed immunohistochemistry on day 36 samples and observed some vesicles that were ISL$^+$ but not PAX8$^+$ (F, G) and some vesicles that were PAX8$^+$ but not ISL1$^+$.

Table S1. List of the top differentially 100 genes in the comparison of CHIR-treated (UP) vs control (DOWN) in the day 13 otic population (OEPD1, OEPD2, OEP, HC)

Click here to download Table S1

Table S2. List of antibodies used for immunocytochemistry

Click here to download Table S2

Development • Supplementary information

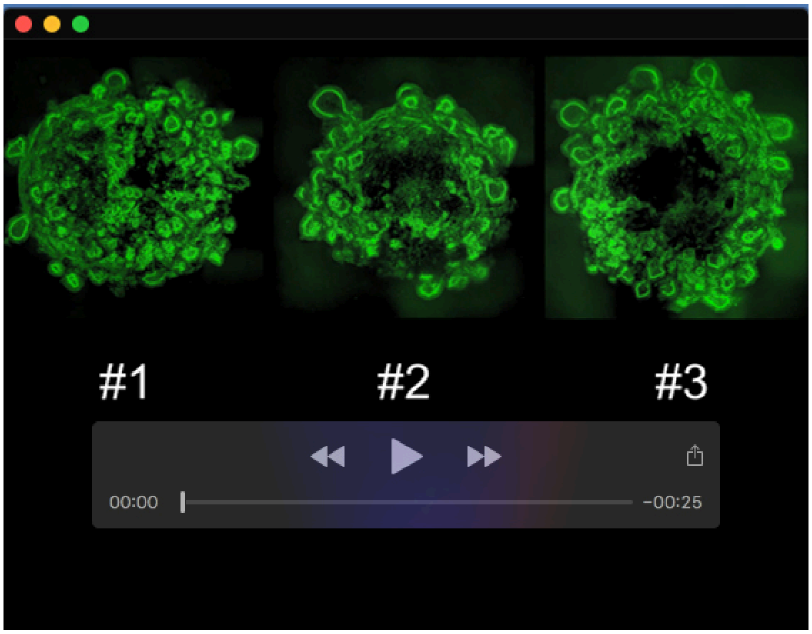

Movie 1. Representative DSP-GFP iPSC-Derived Aggregates Undergoing Inner Ear Organoid Induction. A 24-hour time-lapse recording of three organoids between days 11-12. Z-stacked images were taken every hour. Note the evaginating vesicle-like structures reminiscent of invaginating otic vesicles in the embryo.

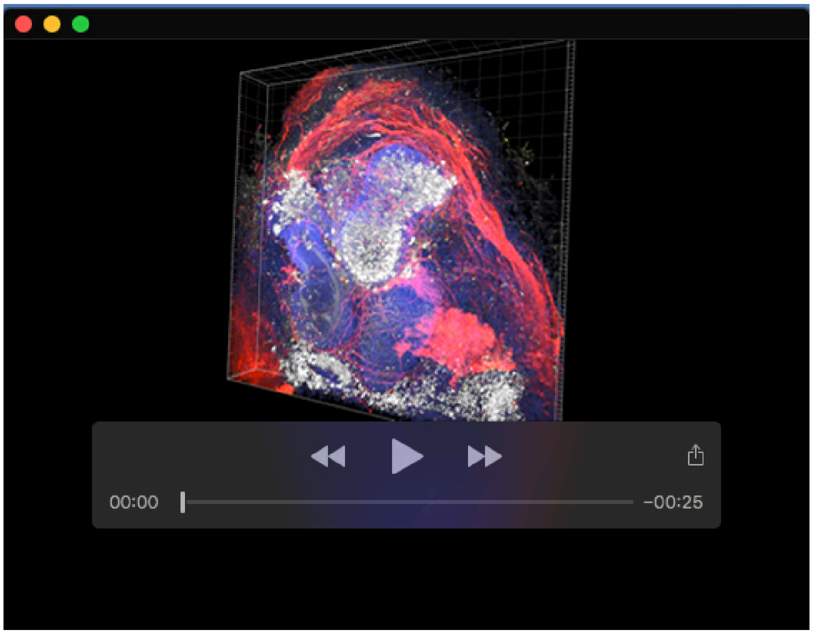

Movie 2. Wholemount Immunostaining of Inner Ear Organoids Derived From DSP-GFP iPSCs. A representative day 80 organoid specimen was wholemount immunostained with antibodies for MYO7A and TUBB3 using a modified SHIELD protocol. The imaged region contains otic organoids with sensory epithelia and dense innervation.

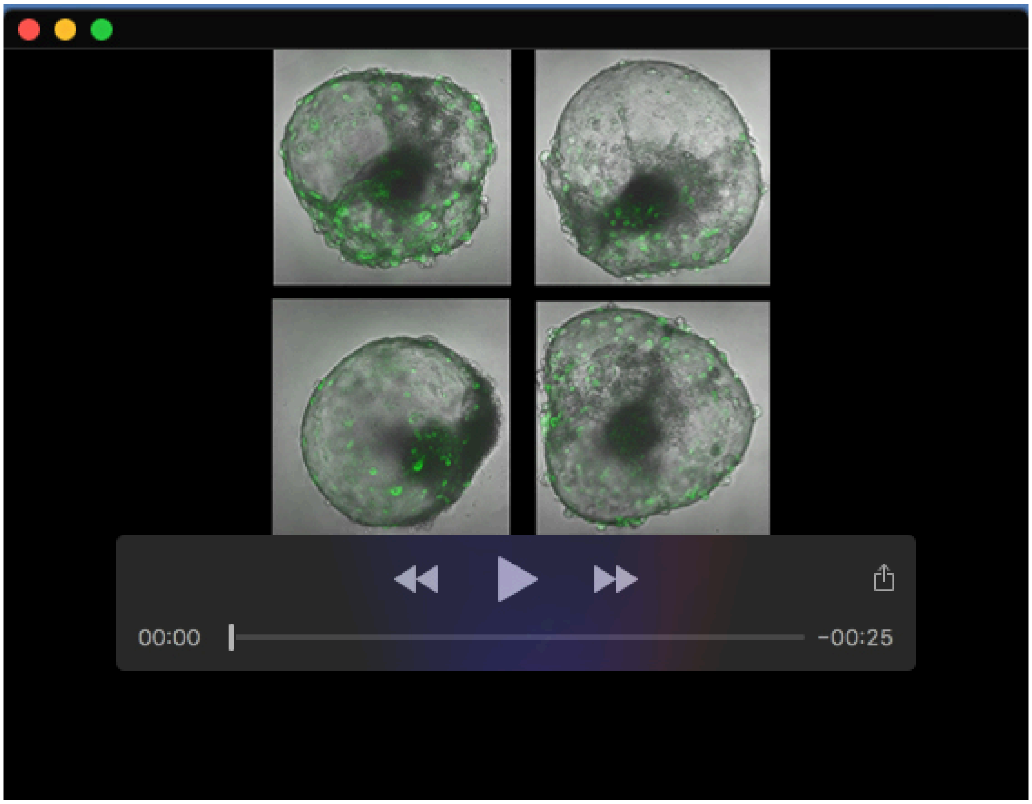

Movie 3. Representative SOX2-GFP iPSC Aggregates Undergoing Inner Ear Organoid Induction. A 24-hour time-lapse recording of three organoids between days 8-9. Z-stacked images were taken every hour. Note the thicken SOX2-GFP-positive patches reminiscent of placodes dotting the surface epithelium.

References

Koehler, K. R., Nie, J., Longworth-Mills, E., Liu, X.-P., Lee, J., Holt, J. R. and Hashino, E. (2017). Generation of inner ear organoids containing functional hair cells from human pluripotent stem cells. *Nat Biotechnol* 35, 583–589.

Ono, K., Sandell, L. L., Trainor, P. A. and Wu, D. K. (2020). Retinoic acid synthesis and autoregulation mediate zonal patterning of vestibular organs and inner ear morphogenesis. *Development* 147, dev192070.

van der Valk, W. H., van Beelen, E. S. A., Steinhart, M. R., Nist-Lund, C., Osorio, D., Groot, J. C. M. J. de, Sun, L., Benthem, P. P. G. van, Koehler, K. R. and Locher, H. (2023). A single-cell level comparison of human inner ear organoids with the human cochlea and vestibular organs. *Cell Rep.* 42, 112623.

Zhang, L., Nomura-Kitabayashi, A., Sultana, N., Cai, W., Cai, X., Moon, A. M. and Cai, C.-L. (2014). Mesodermal Nkx2.5 is necessary and sufficient for early second heart field development. *Dev Biol* 390, 68–79.

Zhang, J., Steinhart, M. R., van der Valk, W. H., Lee, J. and Koehler, K. R. (2021). A simplified method for generating human inner ear organoids from pluripotent stem cells. *Protocol Exchange*.

Development • Supplementary information
