# Supplementary material for: Mapping oto-pharyngeal development in a human inner ear organoid model
Source: Development. 2023 Oct 5;150(19):dev201871. doi: 10.1242/dev.201871 (PMC10698753; doi:10.1242/dev.201871)
Supplement: Supplementary file 1 [file develop-150-201871_review_history.pdf]

Mapping oto-pharyngeal development in a human inner ear organoid model

Matthew R. Steinhart, Wouter H. van der Valk, Daniel Osorio, Sara A Serdy, Jingyuan Zhang, Carl Nist-Lund, Jin Kim, Cynthia Moncada-Reid, Liang Sun, Jiyoon Lee and Karl R. Koehler
DOI: 10.1242/dev.201871

Editor: James Wells

Review timeline

Original submission:	8 May 2023
Editorial decision:	13 June 2023
First revision received:	4 September 2023
Accepted:	8 September 2023

Original submission

First decision letter

MS ID#: DEVELOP/2023/201871

MS TITLE: Mapping oto-pharyngeal development in a human inner ear organoid model

AUTHORS: Matthew R. Steinhart, Wouter H. van der Valk, Daniel Osorio, Jingyuan Zhang, Carl Nist-Lund, Sara A. Serdy, Jin Kim, Cynthia Moncada-Reid, Liang Sun, Jiyoon Lee, and Karl R. Koehler

I have now received all the referees' reports on the above manuscript, and have reached a decision. The referees' comments are appended below, or you can access them online: please go to BenchPress and click on the 'Manuscripts with Decisions' queue in the Author Area.

As you will see, the referees express considerable interest in your work, but have some significant criticisms and recommend a substantial revision of your manuscript before we can consider publication. If you are able to revise the manuscript along the lines suggested, which may involve further experiments, I will be happy receive a revised version of the manuscript. Your revised paper will be re-reviewed by one or more of the original referees, and acceptance of your manuscript will depend on your addressing satisfactorily the reviewers' major concerns. Please also note that Development will normally permit only one round of major revision. If it would be helpful, you are welcome to contact us to discuss your revision in greater detail. Please send us a point-by-point response indicating your plans for addressing the referee’s comments, and we will look over this and provide further guidance.

Please attend to all of the reviewers' comments and ensure that you clearly highlight all changes made in the revised manuscript. Please avoid using 'Tracked changes' in Word files as these are lost in PDF conversion. I should be grateful if you would also provide a point-by-point response detailing how you have dealt with the points raised by the reviewers in the 'Response to Reviewers' box. If you do not agree with any of their criticisms or suggestions please explain clearly why this is so.

© 2023. Published by The Company of Biologists under the terms of the Creative Commons Attribution License (https://creativecommons.org/licenses/by/4.0/).

Reviewer 1

Advance summary and potential significance to field

The scRNA-seq dataset is robust in terms of cell types, timepoints and lineages. As a resource, it could be useful to study many questions about differentiation of neuroepithelial tissues and derivatives, otic and otherwise. The data appear to be accessible in GEO and also in umgear.org, which has a userbase in the inner ear development field.

Comments for the author

Although I am enthusiastic about the resource and many aspects of the manuscript are excellent, I recommend revision first because some of the interpretations in the current Results haven't been substantiated (see Fig. 1H comment below), which could misguide and diminish the utility of the data. The major issue is the argument that these organoids recapitulate otic development in vivo. My assessment of the Results presented is that they do not, and are potentially novel and significant as such. According to the included Response, the authors have a bioarxiv paper titled "Human pluripotent stem cells-derived inner ear organoids recapitulate otic development in vitro" also under review in "Development." Making a conclusion contrary to that may be difficult but nothing I noticed in the preprint supports the otic trajectory claimed here either.

To reconcile the figures with the text, what are needed are 1) a fresh description in Results of the UMAP and trajectories that is supported by the data and not constrained by dogma from in vivo lineage tracing and 2) according interpretations in Discussion. The figures presented imply that factors in this system confer significant otic potential to PSCs to drive OEP generation which is insightful. Not every step in development is likely to be recapitulated in reduced organoid systems nor does it need to be for systems like this to be useful to study differentiation and to generate otic cells.

Fig.1H: The switchback in the OE trajectory (PSC2 – cSE – pPPE – OEPD) is not supported by the Velocity analysis. Neither Fig. S1 nor the subset analysis in Fig. 3 have arrows from pPPE to OEPD. PSCs appear to persist in later time points and the Velocity arrows indicate a direct trajectory from PSC2 to the adjacent OEPD cluster. A simpler interpretation is that the treatments prime remaining PSC2s to differentiate directly into OEPD.

Fig. 1, S1 Arrows in the Velocity plots are too small to evaluate in print.

Fig.1: Have glial cells been removed or grouped into another cluster? The introduction mentions that glia are present.

Is the OEPD – Myocyte trajectory possibly a known lineage (e.g., second heart field) or do they pop up in later timepoint because there are still PSCs after SB/BMP4 washout? Do the myocytes exhibit markers of cardiac or smooth muscle?

Fig. 1H is overplotted and the contributions of individual samples to the dataset are obscured by one another. A figure showing UMAP for each sample relative to the full dataset is needed.

Fig.1i: gene names and cell types are too small to read in print.

The figures throughout are too compressed and have lost resolution needed to read many of smaller labels. Some can only be read by zooming in on the PDF.

Fig. 2B: Some arrows are not supported by the UMAP and Velocity analysis (e.g., NE->NCC) and seem speculative, not inferred as stated.

Fig 2C: please specify the timepoints included

Fig. 3A: Is the D6 sample treated already exposed to FGF/LGN? There appears to be a significant OEPD cluster already by D6.

© 2023. Published by The Company of Biologists under the terms of the Creative Commons Attribution License (https://creativecommons.org/licenses/by/4.0/).

Fig. 3B Velocity arrows are too small to see in print

Fig. 3C, D, G: Subsetting and redoing UMAP in Fig. 3 is not justified. The Velocity data do not support a PSC2 – cSE – pPPE – OEPD trajectory. There is no basis in Fig. 1 UMAP or Velocity data for analyzing NCC as part of a subset with NE and neurons: the NCC are associated with mesenchyme rather than neurons and NE in the main UMAP and there are no arrows suggestive of any trajectory. In any case the expression patterns could be shown on the main UMAP instead of the subset.

Fig. 5A: Velocity does not support the trajectories in the diagram going from OEP, OEPD or SE to the neurons.

Fig. 5C: specify the clusters included in the analysis

Fig. 5D: Velocity in Fig. 1 and Fig. S1 does not support the trajectories diagrammed in D.

Fig. 5F: scales need to be adjusted to see Fgf8 and Pax2 in print.

Fig. 6 Does the otic epithelium show vestibular or cochlear markers e.g., Tectb?
Zpld1?
Is there evidence of hair cell maturation across timepoints (e.g., decreased Atoh1, Sox2)?

Fig. S3A, Fig. S6B: Specify clusters included

Fig. S7A, S8: recompose using the original files. Black boxes are covering some image signal. ˜

Typos:
Abstract: handful of accessory…
Results: we evaluated MKI67 expression, among…
Results: organoid generation from a mixed population…
Figure 2 legend: the presence of ectodermal lineages in the inner…

Reviewer 2

Advance summary and potential significance to field

Studies in this manuscript use state of the art experimental designs such as single cell RNA sequencing and live cell imaging to better characterize inner ear organoids. Diverse tissue subtypes are identified and tracked through key developmental timelines. The validation of tissue subtypes across the differing timepoints is extensively explored and validated through a variety of orthogonal methods from a priori data and complex computational transformations.

Strengths:

1. Extensive thought was placed on validation of organoid tissue with known gene markers and prior knowledge and expands on previous reports on generation of inner ear organoids.

2. This work provides an annotated single cell dataset of IODA- a reference map, as a tool for future inner ear organoid generation.

3. Although it doesn't provide broad new biological insight, a well curated single cell dataset is an important step to understand how to utilize these cultures in the future.

Overall this work provides significant advances in the development of inner ear organoids and provides key benchmarks for the design of future studies.

© 2023. Published by The Company of Biologists under the terms of the Creative Commons Attribution License (https://creativecommons.org/licenses/by/4.0/).

Comments for the author

Overall this is a comprehensive study with clearly presented data and analytic outcomes, likely to be a valuable resource. However the description of data analysis methods is lacking detail. In its current form the analysis is not reproducible. Typically reported parameters such as number of highly variable genes, nearest neighbors for graph analysis etc. are not included. Perhaps the scripts, when posted on GitHub, will provide these details but currently these are not accessible.

There is a discrepancy in the number of PCAs used for the UMAP integration and clustering: main text and supplement appear not to be in accord on this. If I understand correctly, 10 PCs were used for data integration but 100 PCs were used for clustering? This would require further explanation and justification.

More explicit detail is needed on how quality control and filtering was performed. In particular the day 0 dataset is an outlier for features and mt genes. Were cutoffs applied first to individual datasets or to merged? The methods section implies the former but the supplemental data section implies the latter.

Description of RNA velocity analysis is lacking, including parameters used. It doesn't look like results are consistent when clusters are subsetted. For example the vectors appear to be different between FigS1C''' and Figure 3B.

Reviewer 3

Advance summary and potential significance to field

The manuscript by Steinhart et al. provides the results of a single cell RNAseq analysis of the early stages of human inner ear organoid derivation. The resulting data set, which has been made available through gEAR, should be very useful for future work, in particular as a comparator for new manipulations. The authors were very response to previous reviews so I have only a few additional comments.

Comments for the author

Page 8: this is confusing, so if Monocle 3 failed to create a trajectory, how was the trajectory created? Just based on assumed/inferred relationships? Or based on RNA velocity? Were any other trajectory methods tried?

Page 13: while CHIR treatment upregulates Wnt target genes, GSK3 has been shown to modulate other signaling pathways as well. It would be worth looking at target genes for the Notch, Hedgehog and Tgfb pathways also.

Page 15 states that neurons were derived from 4 different precursor clusters, how is this conclusion reached? Several of the presumed lineage relationships illustrated in panel 5D look like very large jumps with no transitional cells present.

Figure 1G, in the UMAP, one cluster is labeled as “otic epithelia” and “OEP” but on the legend, the listing is “Otic epithelium” and “OP”.

© 2023. Published by The Company of Biologists under the terms of the Creative Commons Attribution License (https://creativecommons.org/licenses/by/4.0/).

First revision

Author response to reviewers' comments

Dear Reviewers:

Thank you for thoroughly reviewing our manuscript. We have incorporated your constructive comments and provided additional bioinformatic analysis. Changes in the text are colored blue. Also, recall that we had two other complementary manuscripts under review during our previous submission. The first, Van der Valk et al., was recently published online in *Cell Reports* (link below). The second, Doda et al., was accepted for publication at *Development* and posted online.

Valk et al.: https://www.cell.com/cell-reports/fulltext/S2211-1247(23)00634-4

Doda et al.: Human pluripotent stem cells-derived inner ear organoids recapitulate otic development in vitro | bioRxiv

For our revision analysis, we identified an issue with how the "overview" UMAP plot was generated. If you recall, Figure 4 compared CHIR-treated/untreated samples on days 10 and 13. The ground truth overview plot, referenced throughout the manuscript, should have included the CHIR-treated D10 and D13 datasets. However, during our reanalysis, the untreated D10 and D13 datasets were mistakenly used.

This error occurred during the reanalysis of data from our preprint. The preprint data was prepared correctly with the CHIR-treated datasets, albeit with less stringent quality control filters. Also, the Figure 4 comparative data in the previous version of the manuscript was a true comparison between the CHIR-treated/untreated samples.

We had to rerun the code and regenerate all the plots to rectify the issue. Fortunately, this issue has had minimal impact on the overall conclusions and findings of the paper; however, some clusters are not as closely associated as in the previous version (e.g., OEP and HC). Most changes are aesthetic, as the relative positions of the cell clusters have shifted, and 3 additional clusters have been added. We are relieved to have caught this before publication. We hope the change in figures is not too detrimental to your re-review.

Reviewer 1 Summary:

The scRNA-seq dataset is robust in terms of cell types, timepoints and lineages. As a resource, it could be useful to study many questions about differentiation of neuroepithelial tissues and derivatives, otic and otherwise. The data appear to be accessible in GEO and also in umgear.org, which has a user base in the inner ear development field.

Response: Thank you for these encouraging comments on our work.

Reviewer 1 Comments:

Although I am enthusiastic about the resource and many aspects of the manuscript are excellent, I recommend revision first because some of the interpretations in the current Results haven't been substantiated (see Fig. 1H comment below), which could misguide and diminish the utility of the data. The major issue is the argument that these organoids recapitulate otic development in vivo. My assessment of the Results presented is that they do not, and are potentially novel and significant as such. According to the included Response, the authors have a bioarxiv paper titled "Human pluripotent stem cells-derived inner ear organoids recapitulate otic development in vitro" also under review in "Development." Making a conclusion contrary to that may be difficult but nothing I noticed in the preprint supports the otic trajectory claimed here either.

To reconcile the figures with the text, what are needed are 1) a fresh description in Results of the UMAP and trajectories that is supported by the data and not constrained by dogma from in vivo lineage tracing and 2) according interpretations in Discussion. The figures presented imply that factors in this system confer significant otic potential to PSCs to drive OEP generation, which is insightful. Not every step in development is likely to be recapitulated in reduced organoid systems

© 2023. Published by The Company of Biologists under the terms of the Creative Commons Attribution License (https://creativecommons.org/licenses/by/4.0/).

nor does it need to be for systems like this to be useful to study differentiation and to generate otic cells.

Response: Thank you for providing these thorough comments. We have carefully reviewed your feedback and made improvements to our work. We performed additional analyses to address your points, ensuring a more comprehensive and robust evaluation of our findings. Importantly, we have better highlighted the ambiguities in the data regarding the otic trajectory. We did not intend to be as definitive about this trajectory in the previous version of the manuscript. The path from cSE→pPPE→OEPD1/2→OEP is not fully supported by any one method of trajectory inference. Our new narrative revisions and the new schema in Figure 8 (formerly Fig. 7) more clearly reflect this complexity. We also included a statement, per your suggestion, that it is possible for PSCs to be directly primed for otic induction. Or, perhaps a phenomenon similar to developing cranial neural crest cells is occurring in our in vitro system, whereby cells in the SE populations are transiently reactivating pluripotency transcriptional programs in response to the various treatments (see Zalc et al. 2021).

1. Zalc, A. *et al*. Reactivation of the pluripotency program precedes formation of the cranial neural crest. *Science* **371**, (2021).

In response to your suggestion, we have also enhanced the visibility of our figures. Figure 1 has been split into two figures, Fig 1 covering culture images and Fig 2 covering the initial IODA analysis.

Fig.1H: The switchback in the OE trajectory (PSC2 – cSE – pPPE – OEPD) is not supported by the Velocity analysis. Neither Fig. S1 nor the subset analysis in Fig. 3 have arrows from pPPE to OEPD. PSCs appear to persist in later time points and the Velocity arrows indicate a direct trajectory from PSC2 to the adjacent OEPD cluster. A simpler interpretation is that the treatments prime remaining PSC2s to differentiate directly into OEPD.

Note: Now figure 2D.
Response: Thank you for your feedback. We acknowledge the need for improvement in presenting the inferred trajectories and have reworked Fig 1, now Fig 2D, to illustrate well-supported vs speculative connections between cell clusters. We provide our interpretation of the lineage trajectories in schema embedded in Figs. 2, 3, 4, 5, S1, and 8. For each schema, solid arrows indicate trajectories supported by RNA velocity vectors, and dotted lines are speculative trajectories based on *a priori* developmental biology knowledge and a review of the time-based emergence of the cell clusters. To support the Velocity analysis in our revised manuscript, we have implemented additional Monocle3 analyses focused on the overall UMAP and smaller 2-timepoint time windows. By breaking the data down into 2-timepoint chunks, it was clearer to see how cell types arise and disappear over time. In addition, we suspected that the complexity of the combined data might confound these trajectory inference approaches. The velocity and Monocle3 analyses for each 2-timepoint chunk are in a new **Figure S2**.

The Monocle3 approach identified new, sometimes inconsistent, connections between clusters depending on the timepoint pairing, whereas the RNA velocity analysis remained fairly consistent in the paired timepoint or overall plotting. Thus, *we placed greater weight on the more consistent RNA velocity data* when inferring trajectories.

By incorporating these refined analyses, we can now see that the PSC2 vectors point toward PSC1. The PSC1 vectors appear to diverge and aim toward either the NE or cSE populations in the day 0-3 plots. Subsequently, from D3 to D8 the sequence of pPPE and OEPD1/2 emergence is less clear and we have been careful to communicate this uncertainty to the reader in the revise manuscript. We hope you find our analysis more balanced and less dogmatic.
Moreover, as you said, we cannot dismiss the potential contribution of the persistent PSC2 population that extends up to D30 (Figure S1C-D and F). This remark, along with the aforementioned results, are elaborated upon in the initial section of the results section.

Fig. 1, S1 Arrows in the Velocity plots are too small to evaluate in print.

Note: Now Figure 2B.
Response: Thank you, we adjusted the Velocity representation.

© 2023. Published by The Company of Biologists under the terms of the Creative Commons Attribution License (https://creativecommons.org/licenses/by/4.0/).

Fig.1: Have glial cells been removed or grouped into another cluster? The introduction mentions that glia are present.

Response: Glial cells are part of the neural crest cell-like cluster (NCC). We included the gene marker expression of glial cells within the NCC cluster in Figure S5C.

Is the OEPD – Myocyte trajectory possibly a known lineage (e.g., second heart field) or do they pop up in later timepoint because there are still PSCs after SB/BMP4 washout? Do the myocytes exhibit markers of cardiac or smooth muscle?

Response: With the new analysis, we don't have a source for the arising myocyte population. Based on our recent study on late-stage inner ear organoids, published by Van der Valk in 2023 in Cell Reports, we have discovered that the myocytes that develop within the organoids exhibit a genetic signature resembling cranial skeletal myocytes. Skeletal myocytes are typically derived from the mesoderm germ layer; however, in our organoid system, neither *TBXT*+ mesoderm nor endoderm cells appear to be induced. This raises the question of an alternative source for these myocytes, potentially anterior somitic mesoderm or cranial mesoderm—poorly characterized mesodermal subtypes. Somites are transient epithelial structures that undergo epithelial-to-mesenchymal transition (EMT) to give rise to various muscles, such as those found in the tongue and neck (as reviewed by Frisdal in WIREs Developmental Biology, 2014).
Moreover, anterior somites develop in close proximity to the developing pre-placodal and OEPD region, as discussed in Sai's review in Frontiers in Pharmacology, 2015. Another recent study by John Maroni's lab found that anterior somitic mesoderm arises in the absence of *TBXT* expression and shows expression of *PRRX* genes similar to the lateral plate mesoderm and the cyME, fME, and tME populations in our organoid data (Guibentif et al. 2021). In theory, it is possible that the developing surface ectoderm contains cells reminiscent of somite epithelium. Notably, the somite marker *MEOX1* is expressed in the OEPD2 cluster adjacent to the MYO cluster.

1.Guibentif, C. *et al*. Diverse Routes toward Early Somites in the Mouse Embryo. *Dev. Cell* **56**, 141-153.e6 (2021).

Another possibility is that our initial observations could be attributed to the limitations of relying solely on gene expression data and dimensionality reductions, as recently discussed by Chari and Pachter in their preprint on bioRxiv in 2022. To gain a comprehensive understanding of the progenitor cell populations contributing to the emerging myocytes in our inner ear organoid cultures, we anticipate that lineage-tracing experiments will be necessary. We described these hypotheses in more detail in our discussion and the limitations of our study.

Fig. 1H is overplotted and the contributions of individual samples to the dataset are obscured by one another. A figure showing UMAP for each sample relative to the full dataset is needed.

Response: Thank you for this suggestion. We added the UMAP of each sample to Figure S1B.

Fig.1i: gene names and cell types are too small to read in print.

Note: now figure 2E.
Response: Thank you, we adjusted this.

The figures throughout are too compressed and have lost resolution needed to read many of smaller labels. Some can only be read by zooming in on the PDF.

Response: Thank you for your feedback. We have uploaded the figures at a higher resolution, ensuring better image quality. Additionally, we have increased the size of the small labels to enhance readability. We believe these improvements will enhance the overall experience and make the figures easier to understand.

Fig. 2B: Some arrows are not supported by the UMAP and Velocity analysis (e.g., NE->NCC) and seem speculative, not inferred as stated.

© 2023. Published by The Company of Biologists under the terms of the Creative Commons Attribution License (https://creativecommons.org/licenses/by/4.0/).

Response: To further strengthen our findings, we conducted additional Velocity analysis within specific time windows and performed Monocle3 analysis (Fig. S2). These additional analyses provide supplementary evidence for the identified lineages and prompted us to revise our claims in cases where we lacked supporting evidence. With respect to the development of NCC, our analysis did not yield a conclusive developmental trajectory. However, shared FOXD3 expression (a key regulator of NCC fate) between the NE and NCC clusters, as shown in Fig. S5B, indicates the NE as the potential source of NCC. Moreover, we note that the NCC and NE cell clusters are more closely associated at lower PC levels. Refer to the 10 PC plots in our Supplementary Quality Control analysis. Cluster 13/16 represents NCC cells, and Cluster 8 represents NE cells. We suspect the divergent clustering is a byproduct of the upregulation of genes associated with EMT in NCC cells.

Fig 2C: please specify the timepoints included

Note: now figure 3C.
Response: We appreciate your feedback. The evaluation of gene expression took place on the third day of differentiation. We have now included this information in the legend.

Fig. 3A: Is the D6 sample treated already exposed to FGF/LGN? There appears to be a significant OEPD cluster already by D6.

Note: now figure 4.
Response: The aggregates were supplemented with LDN-193189 at a final concentration of 200 nM and FGF2 at a concentration of 50 µg/ml on day 3, following the protocol outlined in the Materials and Methods section. Interestingly, the emergence of the OEPD cluster was indeed observed as early as day 6.

Fig. 3B Velocity arrows are too small to see in print

Note: now figure 4.
Response: Thank you, we adjusted this figure based on your next comment.

Fig. 3C, D, G: Subsetting and redoing UMAP in Fig. 3 is not justified. The Velocity data do not support a PSC2 – cSE – pPPE – OEPD trajectory. There is no basis in Fig. 1 UMAP or Velocity data for analyzing NCC as part of a subset with NE and neurons: the NCC are associated with mesenchyme rather than neurons and NE in the main UMAP and there are no arrows suggestive of any trajectory. In any case the expression patterns could be shown on the main UMAP instead of the subset.

Note: now figure 4.
Response: Thank you for your feedback. We have removed the subsetting and redoing of the UMAP. Instead, we have incorporated the expression patterns onto the main UMAP, as you recommended.

Fig. 5A: Velocity does not support the trajectories in the diagram going from OEP, OEPD or SE to the neurons.

Note: now figure 6.
Response: Thank you for this comment. We understand the confusion created by the arrows, which seem to recapitulate trajectories. The illustration of the UMAP was placed there as a representation of the questions: What is the extent of neuronal diversity in IEO cultures? In other words, can these be identified as otic, neural crest-derived or epibranchial neurons. For simplicity, we removed the arrows from this illustration. See our further discussion below.

Fig. 5C: specify the clusters included in the analysis

Note: now figure 6.
Response: The expression of marker genes was assessed in both the NE, NEP and NEU1/2 clusters at the various time points. We have now included this additional information in the legend.

Fig. 5D: Velocity in Fig. 1 and Fig. S1 does not support the trajectories diagrammed in D.

© 2023. Published by The Company of Biologists under the terms of the Creative Commons Attribution License (https://creativecommons.org/licenses/by/4.0/).

Note: now figure 6.
Response: Thank you for this comment. Here, we noted that the neuronal populations tended to form a unified cluster based on overlapping “generic neuron” gene expression across PNS and CNS neuronal lineages. The linkage between NE/NEP cells and neurons (NEU1/2) seemed to be the strongest, likely due to the inherent overlap of the NE and neuronal gene expression programs. SE-derived (i.e. placode-derived) neurons have a more divergent gene expression program than their origin/precursors; marked by downregulation of SE-markers, such as CDH1, not expressed in the NE. Thus, one would not expect a clear transitional or linker population between the SE-derived neuroblast cells and their epithelial precursors. In an attempt to find clusters of origin for SE-derived neurons, we looked at early markers of neuroblasts that would presumably be upregulated in epithelial cells before complete commitment to a neuronal fate. You are correct that, based on single-cell data alone, we can not conclusively claim that these distantly located populations of epithelial and neuronal cells are related—we did not mean to give that impression. However, in the case of otic and epibranchial neuroblasts, the IHC results support this possibility. We have removed the arrows and softened our claims regarding these data to avoid biasing the reader.

Fig. 5F: scales need to be adjusted to see Fgf8 and Pax2 in print.

Note: now figure 6.
Response: Thank you, we adjusted this by highlighting its expression.

Fig. 6 Does the otic epithelium show vestibular or cochlear markers e.g., Tectb? Zpld1? Is there evidence of hair cell maturation across timepoints (e.g., decreased Atoh1, Sox2)?

Note: now figure 7.
Response: Thank you for these questions. We now included additional analysis of vestibular versus cochlear marker expression in Figure S9 by which we show expression of both vestibular and cochlear marker genes. This is in correspondence with recent published work in our group in which we show that both vestibular and cochlear epithelium is present at later stage inner ear organoids (van der Valk, Cell Reports, 2023). In addition we also provide some gene marker expression of hair cell development, showing decrease of *ATOH1* and *SOX2* over the later time points and increase of more developed hair cell markers (Figure S9). However, these hair cells are not mature yet as evident by the lack of mature hair cell markers. Both these OEP and HC analyses have been added to the results section.

Fig. S3A, Fig. S6B: Specify clusters included

Note: now figures S4 and S7.
Response: For both figures, the expression analysis was conducted across the entire dataset. We have now included this information in the legends.

Fig. S7A, S8: recompose using the original files. Black boxes are covering some image signal.

Note: now figures S8 and S9.
Response: Thank you for this comment. These images had “burned-in” scale bars that we covered with the black boxes. We have now swapped in the original images.

Typos:
Abstract: handful of accessory…
Results: we evaluated MKI67 expression, among… Results: organoid generation from a mixed population…
Figure 2 legend: the presence of ectodermal lineages in the inner…

Response: Corrected, thank you.

<u>Reviewer 2 Summary:</u>
Studies in this manuscript use state of the art experimental designs such as single cell RNA sequencing and live cell imaging to better characterize inner ear organoids. Diverse tissue subtypes are identified and tracked through key developmental timelines. The validation of tissue

© 2023. Published by The Company of Biologists under the terms of the Creative Commons Attribution License (https://creativecommons.org/licenses/by/4.0/).

subtypes across the differing timepoints is extensively explored and validated through a variety of orthogonal methods from a priori data and complex computational transformations.

Strengths:

1. *Extensive thought was placed on validation of organoid tissue with known gene markers and prior knowledge and expands on previous reports on generation of inner ear organoids.*
2. *This work provides an annotated single cell dataset of IODA- a reference map, as a tool for future inner ear organoid generation.*
3. *Although it doesn't provide broad new biological insight, a well curated single cell dataset is an important step to understand how to utilize these cultures in the future.*

Overall this work provides significant advances in the development of inner ear organoids and provides key benchmarks for the design of future studies.

Response: Thank you for these encouraging comments on our work.

Reviewer 2 Comments:
Overall this is a comprehensive study with clearly presented data and analytic outcomes, likely to be a valuable resource. However the description of data analysis methods is lacking detail. In its current form the analysis is not reproducible. Typically reported parameters such as number of highly variable genes, nearest neighbors for graph analysis etc. are not included. Perhaps the scripts, when posted on GitHub, will provide these details but currently these are not accessible.

Response: We have now shared the scripts used for the analyses through our GitHub page (https://github.com/Koehler-Lab/Inner_Ear_Organoid_Developmental_Atlas). We have to apologize for not sharing these scripts with our initial submission.

There is a discrepancy in the number of PCAs used for the UMAP integration and clustering: main text and supplement appear not to be in accord on this. If I understand correctly, 10 PCs were used for data integration but 100 PCs were used for clustering? This would require further explanation and justification.

Response: Thank you for spotting this typo. We have now corrected the text. We selected 100 PCAs for Harmony integration, UMAP low-dimensional representation generation, and spectral clustering analyses.

More explicit detail is needed on how quality control and filtering was performed. In particular the day 0 dataset is an outlier for features and mt genes. Were cutoffs applied first to individual datasets or to merged? The methods section implies the former but the supplemental data section implies the latter.

Response: We have added more detail about our quality control approach to the methods section. We filtered out cells with extreme (very low or very high, out of the 95% CI) library sizes, number of features, and high content of mitochondrial reads (> 10%). Quality control was performed over the merged dataset. Methodology based on this paper: https://academic.oup.com/bioinformatics/article/37/7/963/5896986

There is now congruence between the methods description and the supplementary data section on QC analysis.

Description of RNA velocity analysis is lacking, including parameters used. It doesn't look like results are consistent when clusters are subsetted. For example the vectors appear to be different between FigS1C''' and Figure 3B.

Response: We have updated our description of how we performed the RNA velocity analysis. We now state in the methods that "A loom file was generated using the original velocyto implementation (La Manno et al., 2018), containing both the spliced and unspliced matrices. RNA velocity calculations were performed using all genes and the stochastic model, employing the Velociraptor wrapper of the scVelo Python library (Bergen et al., 2020). Vector fields were

© 2023. Published by The Company of Biologists under the terms of the Creative Commons Attribution License (https://creativecommons.org/licenses/by/4.0/).

generated based on the baseline UMAP/t-SNE representation, with a resolution of 40 arrows per axis. Pseudotime values were also obtained from the velocity calculations.”

<u>**Reviewer 3 Summary:**</u>
The manuscript by Steinhart et al. provides the results of a single cell RNAseq analysis of the early stages of human inner ear organoid derivation. The resulting data set, which has been made available through gEAR, should be very useful for future work, in particular as a comparator for new manipulations. The authors were very responsive to previous reviews so I have only a few additional comments.

Response: Thank you for these encouraging comments on our work.

<u>**Reviewer 3 Comments:**</u>
Page 8: this is confusing, so if Monocle 3 failed to create a trajectory, how was the trajectory created? Just based on assumed/inferred relationships? Or based on RNA velocity? Were any other trajectory methods tried?

Response: See our response to Reviewer 1’s similar comments.

Page 13: while CHIR treatment upregulates Wnt target genes, GSK3 has been shown to modulate other signaling pathways as well. It would be worth looking at target genes for the Notch, Hedgehog and Tgfb pathways also.

Response: Thank you for your comment. The effect of CHIR99021 treatment on the aggregate at specific timepoints and per cell type on multiple pathways, including Notch, Hedgehog, and TGFb, is captured in the dot plots of Figure S4. You can also review the list of up/down-regulated genes on day 13 in Table S1. We have now stated in the narrative some additional signaling mediators that are upregulated in CHIR-treated organoids on day 13, including TGF signaling mediators (*MSX1/2, ID1, ID2, ID4*), Hedgehog (*HHIP*), and Notch (*NRARP, HES1, NOTCH1*).

Page 15 states that neurons were derived from 4 different precursor clusters, how is this conclusion reached? Several of the presumed lineage relationships illustrated in panel 5D look like very large jumps with no transitional cells present.

Response: See our response to a similar comment from Reviewer 1.

Figure 1G, in the UMAP, one cluster is labeled as “otic epithelia” and “OEP” but on the legend, the listing is “Otic epithelium” and “OP”.

Response: Thank you for bringing this to our attention. We have made the necessary adjustments, and the terms have been updated accordingly to "OEP" and "otic epithelium."

<u>Second decision letter</u>

MS ID#: DEVELOP/2023/201871

MS TITLE: Mapping oto-pharyngeal development in a human inner ear organoid model

AUTHORS: Matthew R Steinhart, Wouter H van der Valk, Daniel Osorio, Jingyuan Zhang, Carl Nist-Lund, Sara A Serdy, Jin Kim, Cynthia Moncada-Reid, Liang Sun, Jiyoon Lee, and Karl R Koehler
ARTICLE TYPE: Techniques and Resources Article

I am happy to tell you that your manuscript has been accepted for publication in Development, pending our standard ethics checks.

© 2023. Published by The Company of Biologists under the terms of the Creative Commons Attribution License (https://creativecommons.org/licenses/by/4.0/).
